# Potential Involvement of *NSD1*, *KRT24* and *ACACA* in the Genetic Predisposition to Colorectal Cancer

**DOI:** 10.3390/cancers14030699

**Published:** 2022-01-29

**Authors:** Isabel Quintana, Pilar Mur, Mariona Terradas, Sandra García-Mulero, Gemma Aiza, Matilde Navarro, Virginia Piñol, Joan Brunet, Victor Moreno, Rebeca Sanz-Pamplona, Gabriel Capellá, Laura Valle

**Affiliations:** 1 Hereditary Cancer Program, Catalan Institute of Oncology, Oncobell Program, IDIBELL, Hospitalet de Llobregat, 08908 Barcelona, Spain; iquintana@idibell.cat (I.Q.); pmur@iconcologia.net (P.M.); mterradas@idibell.cat (M.T.); gaiza@idibell.cat (G.A.); mnavarrogarcia@iconcologia.net (M.N.); jbrunet@iconcologia.net (J.B.); gcapella@idibell.cat (G.C.); 2 Centro de Investigación Biomédica en Red de Cáncer (CIBERONC), 28029 Madrid, Spain; 3 Unit of Biomarkers and Susceptibility, Oncology Data Analytics Program (ODAP), Catalan Institute of Oncology, Hospitalet de Llobregat, 08908 Barcelona, Spain; s.garciam@idibell.cat (S.G.-M.); v.moreno@iconcologia.net (V.M.); rebecasanz@iconcologia.net (R.S.-P.); 4 Consortium for Biomedical Research in Epidemiology and Public Health (CIBERESP), 28029 Madrid, Spain; 5 Gastroenterology Unit, Hospital Universitario de Girona Dr. Josep Trueta, 17007 Girona, Spain; vpinol.girona.ics@gencat.cat; 6 Catalan Institute of Oncology, IDIBGi, 17007 Girona, Spain; 7 Department of Clinical Sciences, Faculty of Medicine, University of Barcelona, 08907 Barcelona, Spain

**Keywords:** hereditary cancer, cancer predisposition, hereditary colorectal cancer, polyposis, somatic second hit

## Abstract

**Simple Summary:**

Methods used for the identification of hereditary cancer genes have evolved in parallel to technological progress; however, much of the genetic predisposition to cancer remains unexplained. A new in silico method based on Knudson’s two-hit hypothesis recently identified ~50 putative cancer predisposing genes, but their actual association with cancer has not yet been validated. In our study, we aimed to assess the involvement of these genes in familial/early-onset colorectal cancer (CRC) using different lines of evidence. Our results indicated that most of those genes were not associated with a genetic predisposition to CRC, but suggested a possible association for *NSD1*, *KRT24* and *ACACA*.

**Abstract:**

The ALFRED (Allelic Loss Featuring Rare Damaging) in silico method was developed to identify cancer predisposition genes through the identification of somatic second hits. By applying ALFRED to ~10,000 tumor exomes, 49 candidate genes were identified. We aimed to assess the causal association of the identified genes with colorectal cancer (CRC) predisposition. Of the 49 genes, *NSD1*, *HDAC10*, *KRT24*, *ACACA* and *TP63* were selected based on specific criteria relevant for hereditary CRC genes. Gene sequencing was performed in 736 patients with familial/early onset CRC or polyposis without germline pathogenic variants in known genes. Twelve (predicted) damaging variants in 18 patients were identified. A gene-based burden test in 1596 familial/early-onset CRC patients, 271 polyposis patients, 543 TCGA CRC patients and >134,000 controls (gnomAD, non-cancer), revealed no clear association with CRC for any of the studied genes. Nevertheless, (non-significant) over-representation of disruptive variants in *NSD1*, *KRT24* and *ACACA* in CRC patients compared to controls was observed. A somatic second hit was identified in one of 20 tumors tested, corresponding to an *NSD1* carrier. In conclusion, most genes identified through the ALFRED in silico method were not relevant for CRC predisposition, although a possible association was detected for *NSD1*, *KRT24* and *ACACA*.

## 1. Introduction

Estimates indicate that ~4% to 15% of all tumors, depending on tumor type, are considered hereditary [1], with genetic alterations being the key determinants of cancer development. Methods used for the identification of hereditary cancer genes have evolved in parallel to technological progress. Classical linkage analysis of large pedigrees followed by positional cloning, and the more recent use of high-throughput sequence capture methods and next generation sequencing technologies, have allowed for the discovery of hereditary cancer genes. Uncovering cancer-predisposing genes improves the molecular diagnosis and personalized surveillance of mutation carriers based on the risks associated with the corresponding gene [2,3].

The genetic predisposition to colorectal cancer (CRC) is partially explained by germline pathogenic variants in the mismatch repair (MMR) genes *MLH1*, *MSH2*, *MSH6* and *PMS2*, *APC*, *MUTYH*, *NTHL1*, *MSH3*, *MLH3*, *POLE, POLD1*, *MBD4*, *AXIN2*, *PTEN*, *BMPR1A*, *SMAD4*, *RNF43* and *RPS20*. Despite the efforts made in recent years to identify additional hereditary CRC genes, much of the genetic predisposition remains unexplained [4].

In 2018 Park et al. published a new in silico method (ALFRED, for Allelic Loss Featuring Rare Damaging) that applies the Knudson’s two hit hypothesis to identify putative cancer-predisposing genes, and applied it to approximately 10,000 tumor exomes [5]. Specifically, they performed a pan-cancer analysis in which they measured the enrichment of rare (MAF < 0.1% according to ExAC) damaging (stop-gain, frameshift, canonical slice-site, or missense predicted pathogenic) germline variants in samples with putative somatic loss of heterozygosity (LOH) for a total of 2983 genes carrying at least five rare (predicted) damaging germline variants. The authors identified 13 genes individually enriched for rare (predicted) damaging variants in tumors. Specifically, five of those genes (*BRCA1*, *ATM*, *BRCA2*, *NSD1* and *TPCN2*) were enriched for germline variants in cases compared to controls. They estimated that germline damaging variants in the 13 proposed genes might explain ~2.3% of the tumors included in The Cancer Genome Atlas (TCGA), which includes 17 individual cancer types. In addition to the 13 genes identified at a false discovery rate of 20%, 12 more, including *MLH1*, were identified in the range of 20–50% false discovery rate, and 24 more genes in the range of 50–60%, making a total of 49 candidate genes for cancer predisposition.

Here we aimed to evaluate the actual involvement in CRC predisposition of the genes identified through the ALFRED in silico method.

## 2. Materials and Methods

**Figure 1:**
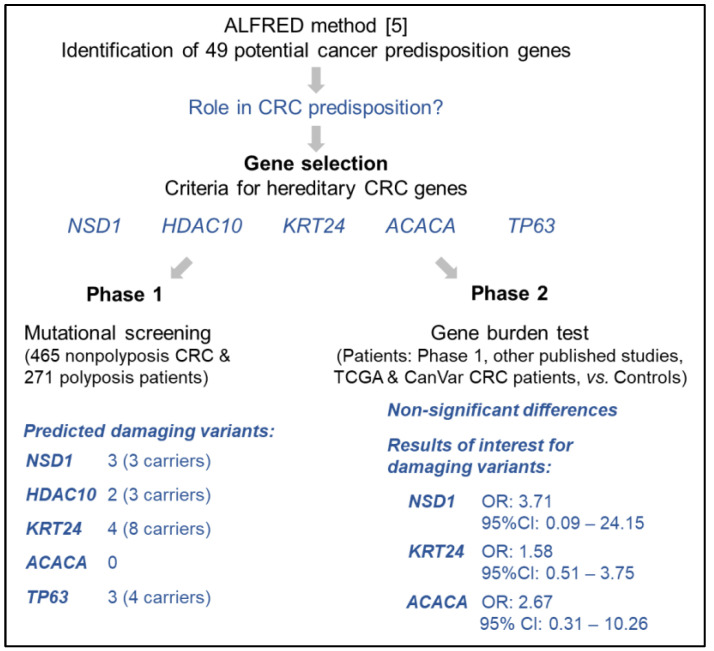
Schematic summary of the study [5].

We selected five of the 49 most enriched genes proposed by Park et al. based on specific criteria considered relevant for CRC predisposition, with the aim of identifying the best candidates for CRC. We next performed mutational screening of the selected genes in 736 unrelated patients with familial/early onset MMR-proficient CRC or polyposis, followed by co-segregation analyses in the relatives of variant carriers. We evaluated the mutational status of the selected genes in additional series of CRC patients with publicly available sequencing data to assess the enrichment of rare damaging germline variants in cases compared to controls (gene burden test). The workflow of the study is summarized in Figure 1.

### 2.1. Patients and Samples

The study included 736 patients (not related, and >99% of European origin): 465 familial/early onset MMR-proficient nonpolyposis CRC patients (Table S1), 177 patients with classic or attenuated adenomatous polyposis (Table S2), and 94 patients with serrated/hyperplastic polyposis (Table S3). The included familial/early-onset nonpolyposis CRC patients had been consecutively recruited through the clinical Hereditary Cancer Program of the Catalan Institute of Oncology (Spain), selected based on the absence of MMR deficiency, assessed by immunohistochemistry and/or microsatellite analysis, and on the absence of germline pathogenic variants in *MUTYH* (biallelic), *NTHL1* (biallelic) or the exonuclease domains of *POLE* and *POLD1*.

Likewise, polyposis patients were consecutively recruited through the same hereditary cancer clinical program, and they were selected for the current study based on the absence of germline pathogenic variants in *APC*, *MUTYH*, *POLE*, *POLD1*, *NTHL1* or *MSH3* in the case of adenomatous polyposis patients, and on the absence of germline pathogenic variants in *RNF43*, *NTHL1* or *MSH3* in the case of serrated polyposis patients [6,7,8,9].

Patients provided written informed consent and the study received the approval of the IDIBELL Ethics Committee (PR073/12).

Genomic DNA from peripheral blood was extracted using the FlexiGene DNA kit (Qiagen, Valencia, CA, USA).

### 2.2. Germline Mutation Identification in Pooled Samples

The abovementioned 736 patients were screened for mutations in *NSD1*, *HDAC10*, *KRT24*, *ACACA* and *TP63* using a combination of PCR amplification in pooled DNAs and targeted next generation sequencing, as previously described [10,11]. Eight DNA pools were generated by adding equimolecular quantities of each sample (48–96 samples per pool). Amplification of the genes’ coding exons (+/− 20 bp flanking regions) was performed in each pool, using Phusion High-Fidelity DNA Polymerase (New England Biolabs, Ipswich, MA, USA) (Primers used are listed in Table S4). Each PCR product was processed as previously described [11,12]. DNA libraries were generated and sequencing at high coverage was performed on a HiSeq-4000 (Illumina, San Diego, CA, USA) at the Centro Nacional de Análisis Genómico (CNAG, Barcelona, Spain). Sequencing data analysis was performed as previously described [11]. The median number of reads per base obtained for all coding regions (+/− 5 bp flanking regions) analyzed was 96,441 (range: 188–420,733 reads/base).

### 2.3. Validation of the Obtained Results and Carrier Identification

Variant-specific KASP genotyping assays (LGC Genomics, Hoddesdon, UK) and direct automated (Sanger) sequencing were used for validation of the targeted next generation sequencing results in the pooled samples, and for identification of the carrier(s) of the corresponding variant (primers in Table S4). Sequencing was performed at STAB VIDA (Caparica, Portugal), and sequencing data were analyzed with SeqMan Pro (Lasergene, DNASTAR, Madison, WI, USA).

### 2.4. In Silico Predictions

Loss-of-function (LoF), canonical splice-site, non-canonical splice-site predicted to alter splicing (Human Splicing Finder [13] version 3.0; http://www.umd.be/HSF3/, accessed on 1 February 2020), and missense variants predicted deleterious by ≥40% of 12 in silico predictors, identified through the pooled-based targeted sequencing approach, were validated by genotyping and/or Sanger sequencing. The 12 in silico prediction tools included SIFT [14], PolyPhen-2 (HVAR and HDIV) [15], MutationTaster [16], MutationAssessor [17,18], PROVEAN [19], LRT [20], MetaSVM [21], MetaLR [21], FATHMM [22], FATHMM-MKL [22], and M-Cap [23]. Prediction data were provided by ANNOVAR [24]. For further analysis, the impact of the validated variants was assessed by the metapredictor REVEL, using a cutoff score for pathogenicity of 0.40; a slightly lower threshold that the one previously established for clinical purposes in known hereditary cancer genes [25,26].

Evolutionary conservation was assessed using PhyloP and PhastCons (obtained from Mutation Taster), based on alignments of genome sequences from 46 different species.

### 2.5. Co-Segregation and Second Hit Analyses

Families carrying disruptive, splice-site, and missense variants predicted deleterious by >40% of the 12 in silico predictors mentioned above were further studied. Sanger sequencing was used to check for the presence of the variant in available samples from relatives. Second-hit analysis in tumors, considering the presence of somatic mutations or loss of heterozygosity, was performed using direct automated (Sanger) sequencing. Sanger sequencing, for either co-segregation or second-hit analysis, was performed at STABVIDA (Caparica, Portugal), and sequencing data analysis was carried out with SeqMan Pro (Lasergene, DNASTAR, Madison, WI, USA).

### 2.6. Gene Burden Test

Results obtained in our study were analyzed in combination with the data obtained from the Cancer Variation Resource (CanVar; https://canvar.icr.ac.uk/) (accessed on 1 September 2021), which include exome sequencing data from 1006 early-onset CRC patients, 863 of whom do not carry germline pathogenic variants in known CRC predisposing genes [27,28].

In addition, blood DNA (germline) exome sequencing data from 543 CRC patients whose tumors are included in the TCGA repository were analyzed. TCGA sequencing data were obtained from NCBI dbGaP (the Database of Genotypes and Phenotypes) after receiving authorization (access request #92142-3). TCGA exomes were analyzed according to the following workflow: FASTQ files were mapped to the reference genome GRCh37/hg19 using the Burrows–Wheeler Aligner (BWA-MEM). Variant calling was performed using the Haplotype Caller (GATK4), results were normalized, and single nucleotide variants and indels were filtered based on the following criteria: read depth < 8, Fisher strand > 25.0, quality by depth < 6.0, and RMS mapping quality < 50.0.

Whenever available, additional gene-specific published results were included in the burden analysis, such being the case for *NSD1* and *ACACA*. TCGA tumor somatic data from the patients with a germline (predicted) damaging variant in the selected genes were obtained via the NCI’s Genomic Data Commons (GDC) platform [29].

For comparison purposes, we used the gnomAD v.2.1.1 non-cancer individuals as control population (*n* = 134,187 individuals; source: (http://gnomad.broadinstitute.org/, accessed on 1 September 2021). Based on the ethnicities of the patients (familial/early-onset CRC and polyposis patients were mostly of non-Finnish European origin; TCGA CRC patients were 51% white, 12% black or African American, 2% Asian, and the other 35% had no information on ethnicity), we decided to repeat the burden tests using the data obtained from the gnomAD v.2.1.1 non-cancer, non-Finnish European subpopulation as controls (*n* = 59,095 individuals).

All genetic variants included in the burden analyses were selected following the exact same criteria: (1) variants with a minor allele frequency (MAF) ≤ 1% according to gnomAD v2.1.1 non-cancer (http://gnomad.broadinstitute.org/, accessed on 1 September 2021); and (2) frameshift, stop-gain, canonical splice-site, start-loss and missense variants predicted damaging (REVEL score > 0.40). The analysis was restricted to allele frequencies.

### 2.7. Statistical Analysis

Gene-based burden tests, i.e., comparison of the frequencies of (predicted) damaging variants in patients and controls, were performed using Fisher’s exact test (two sided). Statistical significance was considered when *p* < 0.01 because five genes were analyzed. Statistical tests and odds ratio (OR) calculations were performed with R version 3.5.1 (RStudio Cloud; RStudio, Boston, MA, USA).

## 3. Results

Figure 1 shows the workflow of the study and a summary of the results obtained.

### 3.1. Gene Selection

With the aim of assessing the actual involvement of the proposed genes in CRC predisposition, we first carried out a pre-selection of the putative cancer predisposing genes identified by Park et al. [5]. To do so, we evaluated the characteristics of the 49 most frequently enriched genes in the original publication based on the following parameters: (i) relevance of the encoded protein in colorectal carcinogenesis; (ii) gene function, focused on relevant hereditary CRC pathways such as DNA repair, Wnt, BMP/TGF-β or mTOR pathways; (iii) expression in normal colon mucosa; (iv) cancer driver gene (https://www.intogen.org, accessed on 1 February 2020); (v) resistance to mutation, measured by a low observed vs. expected ratio of loss-of-function (LoF) variants in control population (source: gnomAD v.2.1.1); and (vi) if the frequency of loss-of-function variants in controls (*n* = 1609) did not exceed their frequency in familial/early-onset CRC patients (*n* = 1006) (case-control data obtained from Chubb et al. [28]). Considering the mentioned characteristics, five of the 49 genes were selected: *NSD1*, *HDAC10*, *KRT24*, *ACACA* and *TP63*. Table 1 shows the characteristics of the selected genes, and Table S5 highlights the main reasons for exclusion of the remaining 44 genes.

### 3.2. Gene Mutational Screening of Familial/Early-Onset CRC and Polyposis Patients

Mutational screening of the five selected genes was carried out in 736 unrelated patients, including 465 familial/early onset MMR-proficient nonpolyposis CRC patients, 177 patients with classic or attenuated adenomatous polyposis, and 94 patients with serrated polyposis. We identified a total of 12 rare (MAF < 1% according to gnomAD v.2.1) variants (predicted deleterious by >40% of 12 in silico prediction tools) in 18 unrelated probands (Table 2). No carriers of *ACACA* rare predicted damaging variants were detected.

Of note, the variant classification guidelines of the American College of Medical Genetics and Genomics and the Association for Molecular Pathology (ACMG/AMP) were not applied to the identified variants, because the recommendations indicate that they should not be used for the classification of variants in genes without a clear association with the disease [39]. Based on this, the variants listed in Table 2 should all be considered as variants of unknown significance regarding their association with cancer.

**Table 3:** Gene burden analysis for *NSD1*, *HDAC10*, *KRT24*, *ACACA* and *TP63*. Statistical analyses compare the data from the different patients’ groups vs. the controls.

Gene	Cohort or Study	Disruptive Alleles		Disruptive, Splice-Site, Start-Loss, Predicted Pathogenic Missense (REVEL > 0.4)	
		*n*/Total Alleles (%)	OR (95%CI); *p*-Value	*n*/Total Alleles (%)	OR (95%CI); *p*-Value
*NSD1*	Controls (gnomAD non-cancer)	15/268,374 (0.01%)		868/268,374 (0.32%)	
	Familial/EOCRC				
	Zhunussova et al.	0/250 (0.00%)		1/250 (0.40%)	
	Chubb et al.	1/2012 (0.05%)		8/2012 (0.40%)	
	Current study	0/930 (0.00%)		0/930 (0.00%)	
	Subtotal	1/3192 (0.03%)	5.61 (0.13–36.44); *p* = 0.17	9/3192 (0.28%)	0.87 (0.40–1.66); *p* = 0.87
	Polyposis (current study)	0/542 (0.00%)	0.00 (0.00–138.68); *p* = 1	1/542 (0.18%)	0.57 (0.01–3.20); *p* = 1
	TCGA CRC patients	0/1086 (0.00%)	0.00 (0.00–68.98); *p* = 1	2/1086 (0.18%)	0.57 (0.07–2.07); *p* = 0.59
	TOTAL patients	1/4820 (0.02%)	3.71 (0.09–24.15); *p* = 0.25	12/4820 (0.25%)	0.77 (0.40–1.35); *p* = 0.44
*HDAC10*	Controls (gnomAD non-cancer)	303/268,374 (0.11%)		1019/268,374 (0.38%)	
	Familial/EOCRC				
	Chubb et al.	3/2012 (0.15%)		6/2012 (0.30%)	
	Current study	0/930 (0.00%)		0/930 (0.00%)	
	Subtotal	3/2942 (0.10%)	0.60 (0.07–2.20); *p* = 0.78	6/2942 (0.20%)	0.54 (0.20–1.17); *p* = 0.17
	Polyposis (current study)	0/542 (0.00%)	0.00 (0.00–6.08); *p* = 1	3/542 (0.55%)	1.46 (0.30–4.30); *p* = 0.47
	TCGA CRC patients	0/1086 (0.00%)	0.00 (0.00–3.03); *p* = 0.64	3/1086 (0.28%)	0.72 (0.15–2.14); *p* = 0.80
	TOTAL patients	3/4570 (0.07%)	0.58 (0.12–1.72); *p* = 0.50	12/4570 (0.26%)	0.70 (0.36–1.21); *p* = 0.22
*KRT24*	Controls (gnomAD non-cancer)	186/268,374 (0.07%)		1016/268,374 (0.38%)	
	Familial/EOCRC				
	Chubb et al.	2/2012 (0.10%)		5/2012 (0.25%)	
	Current study	1/930 (0.11%)		2/930 (0.22%)	
	Subtotal	3/2942 (0.10%)	1.47 (0.30–4.37); *p* = 0.46	7/2942 (0.22%)	0.63 (0.25–1.30); *p* = 0.29
	Polyposis (current study)	0/542 (0.00%)	0.00 (0.00–9.94); *p* = 1	1/542 (0.18%)	0.49 (0.01–2.73); *p* = 0.73
	TCGA CRC patients	2/1086 (0.18%)	2.66 (0.32–9.77); *p* = 0.18	4/1086 (0.37%)	0.97 (0.26–2.51); *p* = 1
	TOTAL patients	5/4570 (0.11%)	1.58 (0.51–3.75); *p* = 0.26	12/4570 (0.26%)	0.69 (0.36–1.22); *p* = 0.27
*ACACA*	Controls (gnomAD non-cancer)	43/268,374 (0.02%)		988/268,374 (0.37%)	
	Familial/EOCRC				
	Thutkawkorapin et al.	0/102 (0.00%)		1/102 (0.98%)	
	Chubb et al.	1/2012 (0.05%)		4/2012 (0.20%)	
	Current study	0/930 (0.00%)		0/930 (0.00%)	
	Subtotal	1/3044 (0.03%)	2.05 (0.05–12.06); *p* = 0.39	5/3044 (0.16%)	0.45 (0.144–1.04); *p* = 0.07
	Polyposis (current study)	0/542 (0.00%)	0.00 (0.00–44.53); *p* = 1	0/542 (0.00%)	0.00 (0.00–1.85); *p* = 0.28
	TCGA CRC patients	1/1086 (0.09%)	5.75 (0.14–33.85); *p* = 0.16	5/1086 (0.46%)	1.25 (0.40–2.94); *p* = 0.61
	TOTAL patients	2/4672 (0.04%)	2.67 (0.31–10.26); *p* = 0.18	10/4672 (0.21%)	0.06 (0.03–0.11); *p* < 2.2 × 10^–16^
*TP63*	Controls (gnomAD non-cancer)	4/268,374 (0.001%)		929/268,374 (0.35%)	
	Familial/EOCRC				
	Chubb et al.	0/2012 (0.00%)		4/2012 (0.20%)	
	Current study	0/930 (0.00%)		3/930 (0.32%)	
	Subtotal	0/2942 (0.00%)	0.00 (0.00–137.82); *p* = 1	7/2942 (0.24%)	0.69 (0.28–1.42); *p* = 0.43
	Polyposis (current study)	0/542 (0.00%)	0.00 (0.00–736.86); *p* = 1	1/542 (0.18%)	0.53 (0.01–2.98); *p* = 1
	TCGA CRC patients	0/1086 (0.00%)	0.00 (0.00–374.35); *p* = 1	0/1086 (0.00%)	0.00 (0.00–0.98); *p* = 0.06
	TOTAL patients	0/4570 (0.00%)	0.00 (0.00–88.76); *p* = 1	8/4570 (0.18%)	0.50 (0.22–1.00); *p* = 0.05

#### 3.2.1. NSD1

Three rare, predicted damaging, germline missense variants were identified in three unrelated probands (3/736 patients). *NSD1* c.3056G>A (p.R1019H) was found in a woman diagnosed with breast cancer and 20 colon adenomas at age 50, and with no family history of cancer. Variant c.3089T>C (p.L1030S) was identified in a patient diagnosed with two CRCs at ages 52 and 59, and with no first-degree relatives affected with cancer. Lastly, *NSD1* c.3151G>A (p.E1051K) was found in: a woman diagnosed with CRC at age 55 and with >70 hyperplastic/serrated polyps; in her sister, who had 16 colorectal polyps at age 61; and in one of her sons, who had two colorectal polyps at age 36. A polyp- and cancer-free son resulted noncarrier. The proband’s father had been diagnosed with bladder and liver tumors at age 72 and 76 respectively, her mother with CRC at age 79, and her maternal grandfather with stomach cancer at 60 years of age. Unfortunately, due to sample unavailability, no co-segregation studies could be performed in those generations. The pedigrees of the carrier families are shown in Figure S1.

#### 3.2.2. HDAC10

Two heterozygous carriers, a priori not related, of the predicted damaging c.308C>T (p.A103V) variant were identified (Pedigrees in Figure S2). One had been diagnosed with breast cancer at age 26, and with CRC and polyps at 35. The other carrier was diagnosed with attenuated polyposis with multiple polyp types at age 63. Neither carrier had relevant cancer family history. *HDAC10* c.827G>A (p.R276G) was present in a man diagnosed with two metachronous CRCs (age at diagnosis: 37 and 43), and with 26 hyperplastic polyps and one adenoma at age 37. His brother and mother were diagnosed with colorectal polyps at 43 and 62 years old, respectively. His cancer family history included one CRC, a prostate cancer, and a pancreatic cancer in two relatives. No co-segregation analysis could be performed.

#### 3.2.3. KRT24

One stop-gain *KRT24* variant, c.130C>T (p.R44*), found in one patient, and three predicted-pathogenic missense variants, found in seven probands, were identified among the 736 patients analyzed. The carrier of the loss-of-function variant had been diagnosed with CRC at age 47 and had family history of various tumor types. *KRT24* c.449G>A (p.R150H) was identified in a woman diagnosed with ovarian cancer at 34 years old, ~40 colonic polyps (adenomas and hyperplastic polyps) at age 40, and CRC at 50. Five probands carried c.1096C>T (p.R366C): four of them had been diagnosed with only CRC (ages 40–69), and the fifth with endometrial cancer and CRC at ages 55 and 77, respectively. The son of one of the carriers, diagnosed with CRC at age 45, also carried the *KRT24* variant identified in his father (CRC, age 69). Lastly, c.1143G>A (p.M381I) was detected in a 35-year-old CRC patient. The pedigrees of the carrier families are shown in Figure S3.

#### 3.2.4. TP63

We identified three rare, predicted damaging, germline variants in *TP63*. Variant c.84T>G (p.H28Q) was identified in two a priori unrelated patients: one diagnosed with CRC at age 50, and another diagnosed with endometrial cancer and CRC at ages 45 and 49, respectively. Both probands had family history of other tumor types. *TP63* c.1127G>A (p.R376H) was identified in a female patient diagnosed with CRC and breast cancer at 56 and 59 years of age, respectively. Her cancer family history included other four CRC cases, four breast cancer cases, and one head and neck cancer identified in her father. *TP63* c.1459C>T (p.R487C) was found in a man diagnosed with five CRCs and 11–20 adenomatous polyps at age 39, with no familial cancer history. Family pedigrees are shown in Figure S4.

### 3.3. Gene Burden Analysis: Assessment of the Association of the Selected Genes with CRC Predisposition

With the aim of elucidating the actual association of germline variants in the selected genes with a predisposition to develop CRC, we compared the frequency of germline damaging and predicted damaging variants in the selected genes in controls (134,187 gnomAD (v.2.1.1) non-cancer individuals) versus the frequency in patients, categorized as: (i) familial and/or early-onset CRC patients (465 from our study, 1006 from Chubb et al. [28]) (source: https://canvar.icr.ac.uk/) (accessed on 1 September 2021), and other reported studies for specific genes); (ii) polyposis patients (271 from our study); and (iii) (mostly) sporadic CRC patients (543 patients from TCGA) (Table 3). For the selection of the variants, we applied a filter that considered variants with a gnomAD non-cancer population MAF below 0.1%, and we used a REVEL cutoff of 0.4, a different, possibly more stringent, value than the criteria used in the discovery phase (>40% of 12 in silico prediction tools). With this criterion, some of the variants listed in Table 2 were not accounted for in the burden test. We applied this cutoff to minimize the inclusion of misclassified, non-damaging missense variants. Since most familial/early-onset CRC and polyposis patients were of non-Finnish European origin, we also performed the analysis considering the gnomAD non-Finnish European subpopulation as controls (Table S7).

Despite the lack of statistically significant differences, *NSD1*, *KRT24* and *ACACA* showed higher frequency of disruptive variants in cases than in controls. This tendency was predominantly observed in familial/early-onset CRC patients for *NSD1*. No association with polyposis was detected for any of the five genes (Table 3).

When comparing the results to non-Finnish European, non-cancer gnomAD individuals as controls, the tendency for *NSD1*, *KRT24* and *ACACA* disruptive alleles remained, and significant association was detected for *KRT24* damaging and predicted damaging variants when comparing TCGA CRC patients to controls (OR = 2.57; 95% CI: 1.35–4.45; *p* = 0.002) (Table S7). Based on the lack of association when comparing to gnomAD non-cancer individuals (OR = 0.97; 95% CI: 0.26–2.51; *p* = 1), it is possible that some of the variants identified in TCGA CRC patients are over-represented in non-European populations, which constitute at least 13% of the TCGA CRC patients analyzed.

### 3.4. Somatic Second Hits

Due to sample availability, we were able to study the presence of acquired somatic mutations or LOH in the selected genes in eight CRCs belonging to *NSD1* and *KRT24* variant carriers (Table 2). No somatic second hits were identified in the CRCs developed by the carriers of *NSD1* p.L1030S and p.E1051K, or in the tumors developed by six of the eight *KRT24* variant carriers, including the CRC of the patient with *KRT24* c.130C>T (p.R44*) (Table S6). Of the 14 carriers of damaging and predicted damaging germline variants in the selected genes identified among the 543 TCGA CRC patients (Table 3), only one, an African American woman diagnosed at age 71 and carrier of the germline variant *NSD1* c.4892A>G (p.K1631R), had a CRC with a somatic mutation in the same gene: c.6143T>A (p.I2048N).

## 4. Discussion

Park et al. devised a statistical method, termed ALFRED, that tests Knudson’s two-hit hypothesis genome-wide to systematically identify cancer predisposition genes from cancer genome data [5]. By applying ALFRED to >10,000 tumor exomes from 30 cancer types, they identified up to 49 putative cancer predisposition genes. This study caught our interest and we decided to test their hypothesis by assessing the role of the identified ALFRED genes in the predisposition to CRC. First, we performed a pre-selection of genes based on different criteria, which led to a shortened list of five genes as the best candidates to be involved in CRC predisposition: *NSD1*, *HDAC10*, *KRT24*, *ACACA* and *TP63*. We identified a total of 12 damaging and predicted damaging variants in 18 probands of a series of 465 MMR-proficient CRC patients and 271 polyposis patients without germline pathogenic variants in known polyposis genes. To demonstrate the association of pathogenic variants in those genes with an increased risk of CRC, we then compared the frequency of damaging and predicted damaging variants in CRC patients and controls, including data from our study as well as others publicly available (publications and databases). Despite the lack of statistical differences between cases and controls, perhaps due to the small number of positive cases, overrepresentation of disruptive (stop-gain and frameshift) variants in cases was observed for *NSD1*, *KRT24* and *ACACA*. No clear association was observed for *HDAC10* and *TP63*.

NSD1 (histone H3 lysine 36 methyltransferase) is involved in chromatin organization and is considered an epigenetic regulator [40]. Somatic loss-of-function *NSD1* mutations are among the most prevalent lesions in human head and neck and lung squamous cell carcinomas, neuroblastomas and glioblastomas, and *NSD1* gene silencing has been detected in clear cell renal cell carcinoma and urogenital cancers (reviewed by Tauchmann and Schwaller [40]). Little is known regarding the role of NSD1 in colorectal cancer; however, publicly available data indicate that *NSD1* somatic alterations occur in 4% of colon cancers (source: cBioPortal; accessed January 2022).

**Figure 2:**
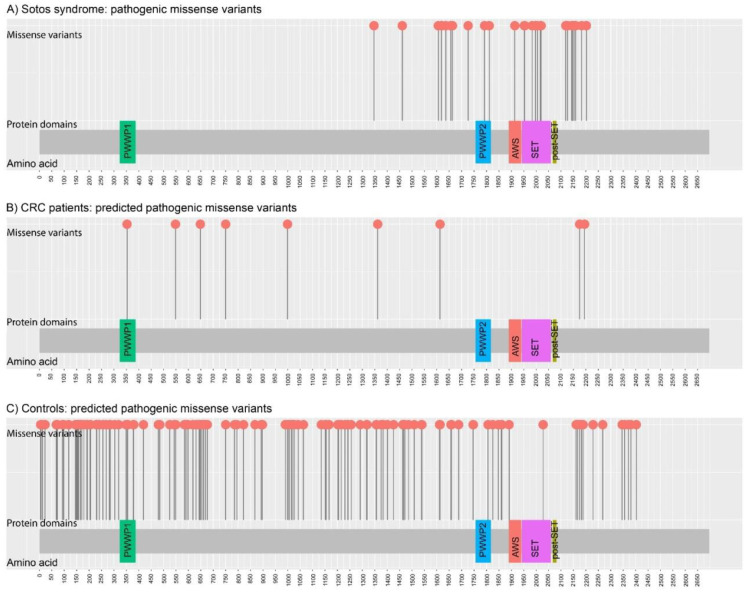
Distribution of germline missense *NSD1* variants identified in: (**A**) Sotos syndrome (*n* = 32 pathogenic missense variants; source: ClinVar; accessed on September 2021); (**B**) CRC patients (*n* = 9 rare predicted damaging (REVEL > 0.4) missense variants; source: current study; Chubb et al. (2016); Zhunussova et al. (2019); and TCGA); and (**C**) Controls (*n* = 138 rare predicted damaging (REVEL > 0.4) missense variants; source: gnomAD (v.2.1.1) non-Finnish European, non-cancer individuals). Figure created with MutPlot [43].

Heterozygous pathogenic variants in *NSD1* are detected in 70% to 93% of typical Sotos syndrome patients [41]. Sotos’ disruptive mutations are spread throughout *NSD1*; however, pathogenic missense mutations related to the syndrome are clustered in highly conserved functional domains between exons 13 and 23 [42]. Looking for clinically validated variants in ClinVar, a total of 246 coding changes have been reported as pathogenic in Sotos syndrome patients: 196 disruptive and 18 canonical splice-site variants are distributed throughout the gene (exons 5 to 23), whereas all missense pathogenic variants (*n* = 32) are located between exons 13 and 23 (Figure 2A), in agreement with the observation by Douglas et al. in 2003 [42]. Despite putatively sharing the same autosomal dominant inheritance, variants identified in CRC patients show different characteristics. While most variants identified in Sotos patients are loss-of-function, only one disruptive variant has been identified in CRC patients (*NSD1* c.7874G>A; p.W2625*), while the others included nine missense predicted pathogenic (REVEL>0.4). Five out of the nine missense variants identified in CRC patients affected exons 5 and 6, and the other four exons 13, 18 and 23 (Figure 2B). In contrast to the exons 13–23 location usually observed in Sotos syndrome, CRC patients showed a more homogeneous distribution of missense pathogenic variants, similar to that observed in controls (Figure 2C). Park et al. had already noticed differences between the missense variants identified in cancer patients compared to those observed in Sotos syndrome patients [5]. While *NSD1* was the second most significantly enriched gene in the study performed by Park et al., our results did not show a clear association with CRC predisposition. Zhunussova et al. analyzed 125 early-onset CRC patients from Kazakhstan with a gene panel that included *NSD1*. Despite their statement that *NSD1* was one of the most mutated genes (399 variants in the 125 patients), only one variant, *NSD1* c.1865G>C (p.C622S), had a REVEL score > 0.4 [31]. In fact, in addition to the pan-cancer association, this gene was found to be particularly associated with ovarian, stomach, bladder, lung and liver cancers, but not specifically with CRC in the original ALFRED publication [5].

*KRT24* encodes a keratin essential for the cytoskeleton of epithelial cells and it also influences cellular response to pro-apoptotic signals. Our results suggested that disruptive variants in *KRT24* were enriched in CRC patients compared to controls (0.11% vs. 0.07%), although the differences did not reach statistical significance. Aside from the pan-cancer association, Park et al. did not identify a particular association for CRC in the case of *KRT24* [5]. Overexpression of *KRT24* has been found in the normal mucosa of early-onset MMR-proficient CRC patients compared to the normal mucosa of healthy controls, supporting its role in CRC predisposition [35], although its association with the presence of germline variants in the gene was not evaluated. Apart from our analysis, no other published studies include the study of germline variants in *KRT24* in CRC patients. Somatic mutations in *KRT24* occur in 1.6% of CRCs (source: cBioPortal; accessed January 2022).

ACACA (acetyl-CoA carboxylase or ACC1) is a key catalyzer in the biogenesis of long-chain fatty acids, which are essential for cancer cell survival during hypoxia [44]. Inhibition of *ACACA* leads to decreased cell proliferation, decreased apoptosis, and increased risk of metastasis or recurrence [45,46,47,48]. While Park et al. identified a pan-cancer association, no specific association with CRC was detected [5]. Our results showed an over-representation of disruptive variants in CRC patients compared to controls, although the difference did not reach statistical significance. Thutkawkorapin et al., by performing exome sequencing in 51 early-onset CRC patients without family history of cancer, identified a missense predicted pathogenic variant in *ACACA*, p.R2208Q, in one of the patients, and proposed it as a candidate for CRC predisposition [36]. To our knowledge, no additional studies of *ACACA* in CRC patients have been published. Somatic *ACACA* mutations occur in 4% of CRCs (source: cBioPortal; date of access: January 2022)

Regarding the other two genes, *HDAC10* and *TP63*, for which no over-representation of (predicted) damaging variants was identified in CRC patients compared to controls, no previous studies have identified an association with CRC predisposition. *TP63* alleles have been associated with susceptibility to different cancer types, but not to CRC (some examples: [49,50,51,52,53]). Somatic mutations in *HDAC10* and *TP63* occur in 1.1% and 2.4% of CRCs respectively (source: cBioPortal; accessed January 2022).

Considering the rationale behind the ALFRED in silico method, we assessed the presence of somatic second hits (somatic variant or LOH) in available tumor samples from the carriers identified in our series (two tumors from *NSD1* variant carriers and six tumors from *KRT24* variant carriers), and from the 14 TCGA CRC patients with a damaging or predicting damaging variant in any of the five selected genes. Only one of the 20 CRCs had an acquired somatic mutation in the corresponding gene, which corresponded to a tumor from an *NSD1* variant carrier. Somatic methylation was not evaluated.

The major methodological limitations of our study include: (i) Sample sizes for the burden tests were insufficient, which may have prevented the identification of significant associations. Due to the extremely low prevalence of disruptive variants, larger series of patients need to be analyzed, adding complete co-segregation and second hit analyses. (ii) The gene pre-selection step might have excluded one or several relevant genes for CRC predisposition. In this regard, future studies should not systematically discard the remaining 44 ALFRED genes as potentially involved in CRC predisposition. (iii) Moreover, based on the lack of studies that functionally link alterations in those genes with colorectal carcinogenesis, functional studies that prove their role in the initiation of (colorectal) cancer will also be key for their confirmation as CRC predisposition genes. (iv) While our study covers CRC, most ALFRED genes might be involved in the predisposition to other tumor types, such as ovarian, breast and endometrial cancers, as had been proposed in the original publication [5].

## 5. Conclusions

Aiming to assess the involvement in CRC predisposition of previously identified potential cancer predisposition genes (ALFRED genes), we performed a mutational screening of five selected genes in 736 familial/early-onset CRC and polyposis patients, followed by gene burden analyses that compared the frequency of damaging and predicted damaging variants in CRC patients and controls. Our study showed that all, or at least most, ALFRED genes did not seem to be relevant for CRC predisposition, at least not as monogenic cause of the disease and/or following a classic tumor suppressor model (Knudson’s second hit hypothesis). Nevertheless, the results obtained in our study, although nonsignificant probably due to insufficient sample size, suggest a possible association of *NSD1*, *KRT24* and *ACACA* disruptive (loss-of-function) variants with CRC, requiring validation in larger series of patients.

## Figures and Tables

**Table 1:** Characteristics of the selected candidate genes (identified using the statistical method ALFRED by Park et al. [5]).

Gene	Function	Role in CRC	Previous Association with Cancer Predisposition	^a^ Expression in Normal Colon Mucosa	Significantly Enriched Cancer Type by ALFRED	^b^ Cancer Driver Gene (Tumor Type)	^c^ Observed vs. Expected LoF Variants (gnomAD)	^d^ Disruptive Variants in Cases vs. Controls. (Chubb et al.)	Syndromes Caused by Germline Mutations (Mode of Inheritance)
*NSD1*	Negative regulation of RNApol II transcription. Regulation of histone H3K36 methylation.	*NSD1* expression is a marker of poor prognosis [30].	Germline *NSD1* c.1135G>A (p.A379T) identified in an early-onset CRC patient without family history of cancer [31].	Yes	Pancancer (FDR 20%), BLCA, LIHC, LUSC, OV, STAC.	Yes (HNSCC, EC, LUSC, ESC, CSC, STAC, UCS)	5/110.6	Cases: 0 Controls: 0	Sotos syndrome (AD)
*HDAC10*	Chromatin organization, transcriptional regulation, cell cycle progression and DNA repair.	Wnt pathway regulator in CRC cell lines [32]. Possible tumor suppressor function in CRC [33]. Potential involvement in DNA mismatch repair [33,34].	No	Yes	Pancancer (FDR 20–50%)	No	43/36.8	Cases: 1 Controls: 0	None
*KRT24*	Organization of membrane proteins. Apoptotic cellular response.	None reported.	Overexpressed in normal mucosa of early-onset CRC patients [35].	Yes	Pancancer (FDR 50–60%), OV.	No	24/23.3	Cases: 2 Controls: 0	None
*ACACA*	Cell energy maintenance (fatty acid biosynthesis). Cell proliferation control.	None reported.	Germline c.6623G>A (p.R2208Q) identified in an early-onset CRC patient without family history of cancer [36].	Yes	Pancancer (FDR 50–60%)	No	20/134.5	Cases: 1 Controls: 0	Acetyl-CoA carboxylase deficiency (AR)
*TP63*	Development, stem cell regulation, premature aging, and DNA damage response. WNT negative regulator.	*TAp63* expression is downregulated in CRC [37]. Expression of *TP6*3 is a prognostic marker [38].	No	Yes (low)	Pancancer (FDR 50–60%)	Yes (EC, BLCA, HNSCC, NB)	4/19.5	Cases: 0 Controls: 0	Various developmental syndromes with craniofacial and skeletal abnormalities (AD) [OMIM 603273]

^a^ Data obtained from www.colonomics.org (accessed on 1 February 2020). Samples: 100 stage II untreated colon tumors, 100 normal paired colon mucosa and 50 normal colon mucosa obtained from healthy donors, ^b^ Information obtained from www.intogen.org (accessed on 1 February 2020), ^c^ Source: GnomAD v.2.1, non-Finnish European, non-cancer subpopulation. ^d^ Chubb et al. study includes 1006 CRC cases and 1609 controls. Disruptive variants are defined as nonsense and frameshift. Abbreviations: AD, autosomal dominant; BLCA, bladder cancer; CRC, colorectal cancer; CSC, cervix squamous cancer; EC, endometrial cancer; ESC, esophageal cancer; HNSCC, head and neck squamous cell carcinoma; LoF, loss of function; LIHC, liver hepatocellular carcinoma; LUSC, lung squamous cell carcinoma; NB, neuroblastoma; OV, ovarian cancer; STAC, stomach adenocarcinoma; UCS, uterine carcinosarcoma.

**Table 2:** Novel and rare (MAFgnomAD < 1%) germline variants predicted deleterious by ≥40% of 12 in silico tools, identified in 736 familial/early-onset MMR-proficient CRC or polyposis patients. Phenotypic data from the probands and relatives, together with co-segregation results, are detailed in Table S6 and Figures S1–S4 (pedigrees).

Gene (Transcript)	Family ID	Variant	dbSNP	^a^ Population MAF%	^b^ In Silico Prediction (REVEL Score)	^c^ Evolutionary Conservation (PhyloP/Phast-Cons Scores)
*NSD1* (NM_022455)	F1	c.3056G>A (p.R1019H)	rs750354456	0.00195	0.416 (D)	3.876/1.000
	F2	c.3089T>C (p.L1030S)	rs200856103	0.04579	0.365	2.905/1.000
	F3	c.3151G>A (p.E1051K)	rs141014337	0	0.329	3.287/1.000
*HDAC10* (NM_001159286)	F4, F5	c.308C>T (p.A103V)	rs143228101	0.03606	0.658 (D)	9.957/1.000
	F6	c.827G>A (p.R276G)	rs752737416	0.00186	0.6179 (D)	1.6579/0.987
*KRT24* (NM_019016)	F7	c.130C>T (p.R44*)	rs148493418	0.02725	-	-
	F8	c.449G>A (p.R150H)	rs146614779	0.00762	0.880 (D)	6.124/1.000
	F9, F10, F11, F12, F13	c.1096C>T (p.R366C)	rs16966138	0.05585	0.514 (D)	2.990/1.000
	F14	c.1143G>A (p.M381I)	rs375745897	0.01523	0.515 (D)	7.501/1.000
*TP63* (NM_003722)	F15, F16	c.84T>G (p.H28Q)	rs370716448	0.00509	0.449 (D)	1.792/1.000
	F17	c.1127G>A (p.R376H)	rs143591434	0.00195	0.495 (D)	7.106/1.000
	F18	c.1459C>T (p.R487C)	rs777306829	0.01696	0.636 (D)	3.485/1.000

^a^ GnomAD v2.1. non-Finnish European (NFE), non-cancer individuals. ^b^ REVEL cutoff score considered for deleteriousness (D): >0.40. Predicted damaging variants were included in the burden tests (Table 3). ^c^ PhyloP score range: −20, +10; positive values indicate conserved residues. Phast-Cons score range: 0, 1; higher values (closer to 1) indicate conserved positions.

## Data Availability

All data supporting the reported results may be found in the article and supplementary material.

## Supplementary Materials

The following supporting information can be downloaded at: https://www.mdpi.com/article/10.3390/cancers14030699/s1, Table S1: Characteristics of the 465 MMR-proficient non-polyposis unrelated CRC patients included in the study, Table S2: Characteristics of the 177 unrelated adenomatous polyposis patients included in the study, Table S3: Characteristics of the 94 serrated polyposis patients included in the study, Table S4: Amplification and sequencing primers used for Sanger and targeted next generation sequencing, Table S5: Reasons for exclusion for the other 44 genes identified by Park et al., Table S6: Clinical characteristics, co-segregation data and second hit results of the carriers of the rare, predicted damaging variants identified among the 736 patients included in the study, Table S7: Burden analysis of the germline variants identified in the five selected candidate genes, considering the gnomAD non-Finnish European (NFE), non-cancer subpopulation as controls, Figure S1: Pedigrees of the families carrying NSD1 rare germline (predicted) damaging variants, Figure S2: Pedigrees of the families carrying HDAC10 rare germline variants, Figure S3: Pedigrees of the families carrying KRT24 rare germline variants, Figure S4: Pedigrees of the families carrying TP63 rare germline variants.
