# Supplementary material for: Potential Involvement of NSD1, KRT24 and ACACA in the Genetic Predisposition to Colorectal Cancer
Source: Cancers (Basel). 2022 Jan 29;14(3):699. doi: 10.3390/cancers14030699 (PMC8833793; doi:10.3390/cancers14030699)
Supplement: Supplementary file 1 [file cancers-14-00699-s001.zip › cancers-1579787-supplementary.pdf]

cancers

MDPI

SUPPLEMENTARY MATERIAL

Table S1. Characteristics of the 465 MMR-proficient non-polyposis unrelated CRC patients included in the study.

	n	Mean Age at Cancer Diagnosis	Median Age at Cancer Diagnosis	Age Range
**Proband affected with CRC**				
Amsterdam [I / II]	59 [43/15]	46.08	46	32-78
Bethesda	378	45.95	47	16-82
No HNPCC criteria	24	40.35	40	37-44
Subtotal	461	45.88	47	16-82
**Proband affected with other tumor types**				
Amsterdam [I / II]	3 [0/3] [a]	44.13	44	39-51
Bethesda	1 [b]	47	47	47
Subtotal	4	44.81	45.5	39-51
**TOTAL**	**465**	**45.87**	**47**	**16-82**

[a]Two probands diagnosed with gastric cancer at ages 51 and 39, respectively, and one proband diagnosed with urethral cancer at age 44). [b]Proband diagnosed with endometrial cancer at age 47. Abbreviations: CRC, colorectal cancer; HNPCC, hereditary nonpolyposis colorectal cancer.

Table S2. Characteristics of the 177 unrelated adenomatous polyposis patients included in the study.

Polyp Number	# Of Patients (Median Age Polyposis dx; Range)	# Of Patients with CRC (Median Age at 1st CRC Diagnosis; Range)	# Of Patients with Synchronous or Metachronous CRC	# Of Patients with Multiple Primary Tumors (CRC and/or Extracolonic)	# Of Patients with a FDR with Polyposis	# Of Patients with a FDR with CRC
	**177 (56.5; 14-83)**	**106/177 [59.9%] (59; 29-75)**	**33/177 [18.6%]**	**42 [23.7%]**	**39/176 [22.1%]**	**66/176 [37.5%]**
**ADENOMATOUS POLYPOSIS**						
>100 polyps	4 (52.5; 38-58)	3/4 (50; 38-58)	1/4	1/4	1/4	2/4
51-100	19 (56.5; 29-77)	9/19 (61; 29-77)	6/19	6/19	5/19	4/19
21-50	50 (59.5; 29-83)	33/50 (64; 35-76)	14/50	18/50	10/50	25/50
10-20	44 (59; 30-78)	29/44 (60; 30-76)	5/44	8/44	11/43	15/43
<10	6 (51.5; 14-66)	2/6 (64; 63-65)	1/6	1/6	3/6	2/6
unknown polyp #	1 (42)	1/1 (42)	0/1	0/1	0/1	1/1
**SUBTOTAL**	**124 (59; 14-83)**	**77/124 (61; 29-77)**	**27/77**	**34/77**	**30/76**	**49/76**
**POLYPOSIS OF MULTIPLE POLYP TYPES (Ad + HP)**						
>100 polyps	2 (52; 48-56)	2/2 (52; 48-56)	0/2	0/2	1/2	1/2
51-100	8 (54; 41-61)	3/8 (54; 41-65)	1/8	1/8	1/8	3/8
21-50	22 (56; 42-75)	11/22 (56; 44-75)	2/22	3/22	5/22	9/22
10-20	13 (57; 36-63)	8/13 (51.5; 46-59)	1/13	2/13	1/13	3/13
unknown polyp #	1 (49)	1/1 (49)	1/1	1/1	0/1	0/1
**SUBTOTAL**	**46 (55; 36-75)**	**25/46 (54; 41-75)**	**5/46**	**7/46**	**8/46**	**16/46**
**NON-CLASSIFIED POLYPOSIS**						
>100 polyps	1 (40)	0/1	0/1	0/1	1/1	1/1
51-100	1 (49)	1/1 (49)	0/1	0/1	0/1	0/1
21-50	2 (53.5; 53-54)	2/2 (53.5; 53-54)	0/2	0/2	0/2	0/2
10-20	1 (35)	1/1 (35)	1/1	1/1	0/1	0/1
unknown polyp #	1 (36)	0/1	0/1	0/1	0/2	0/2
**SUBTOTAL**	**6 (44.5; 35-54)**	**4/6 (51; 35-54)**	**1/6**	**1/6**	**1/6**	**1/6**
**DUODENAL POLYPOSIS**						
<10 (2 Ad + 2 HP)	**1 (69)**	0/1	0/1	0/1	1/1	**0/1**

Abbreviations: Ad, adenoma; CRC, colorectal cancer; FDR, first degree relative; HP, hyperplastic polyp; #, number.

Table S3. Characteristics of the 94 serrated polyposis patients included in the study.

Age at polyposis diagnosis	Mean age: 51.8 years (range: 8-73)
Total # of polyps	Mean #: 39 (range: 3-195)
# of hyperplastic/serrated polyps	Mean #: 28.6 (range: 3-163)
# of adenomas	Mean #: 3.9 (range: 0-65)
# of patients with histologic report of serrated polyps	Total #: 45 (47.9%)
**Characteristics of the serrated polyposis patients**	**# cases (%)**
Gender	Male: 61 (64.9%)<br>Female: 33 (35.1%)
CRC diagnosis	34 (36.2%)<br>Mean age at diagnosis: 51.9 (range: 24-75)
Family history of polyposis	14 (14.9%)
Cases with adenomas in addition to hyperplastic/serrated polyps	66 (70.2%)<br>Mean # of adenomas: 6 (range:1-65)
APC gene tested	[a]37/94 (39.4%)
MUTYH gene tested	45/94 (47.9%)
RNF43 gene tested	94/94 (100%)

a. Variants of unknown significance in *APC* were detected in 2 individuals.

Table S4. Amplification and sequencing primers used for Sanger and targeted next generation sequencing.

Gene	Exon/s	Forward Primer (5'-3')	Reverse Primer (5'-3')	Size (bp)
NSD1 (NM_022455)	1A	AGGCCACTAGGCCTTGAAGT	ATCTGCATCGTCCACACAAG	597
	1B	TTCCTTGAGTCCTGGTGGTC	TTTCCCTTTAAGTGGCCTGT	653
	2	TGCTTTTTCAGAAGGCTAATAGG	TCATTCACAAAATGTTCCAAGG	332
	3	GCAATGATGTGGCTGTTCTC	TCCAATCTGGGAAACAGAGC	364
	4A	TCTGATTTCATCTCCCTTTTCC	GGCTCTGCACTCTTAGTACAACC	999
	4B	TGTCCTTGAAATTCCAGATGC	TCCGGTAAGAGACCAGGTTTT	830
	4C	TGGAACATCAAAGCCATCAA	CTCCCTGCAGTACAGCATCA	575
	4D	ATGCTTTTTCAGCCCAAATG	TGATTCTTCCCCGCTAGTTT	822
	5	GGGAGTATCAGATGGTCTCATAAAA	TTCATGAAGATAAAGTATGACATTGAA	291
	6	GCCTTTGTCAGAATTTCATTCC	CCAGGACAAAAGGGGGTAGT	499
	7	ATGACGGGGAAAACATCAAA	CAACTGCAAAACAGCCTTTC	399
	8	TGGCAGCTGACAATTCAGAC	TGGTTAACAGACAAAGCACTTCA	233
	9	CCACAAAGCTGGAGAATTAAATG	TCAAGGGAAAGAGGGATGTG	317
	10	AGAGGGAGGGGGTCAAATG	CCATCATAAAGAGATGGAGTGG	291
	11	CCCACTGACACTGGTTTTACTC	CCCAGTGTTGCCACAAAATA	296
	12	TGGGTTCAGACGATGTCAAA	CAGCGAGACTCTGTCTCCAA	438
	13	TGGTCATTCCTTCTATTTGTGA	TTCCAGTGGCAATATGATGAA	399
	14	GCTTATGCTGTTTATACTCTGGTCA	CGTTAAGCATTGTATCTGAGAGG	396
	15	TGTGGACAGACAGACATTGCT	TGGAGCATATGTGAATTCTAAAACA	398
	16	TCTCCAACTTAAAGGGGAAAAA	TTGTGCTTCCGTTCTTGTCA	298
	17	TTTGCCTTTTTCAGGACGTG	CGGTAGAAGGATATGGCAAGA	490
	18	CATTATCAAATAGTTGGGGATCG	CCTTGGGAACTGCGTTACAT	381
	19	CTGGTCCAGGGATCACACTT	AAAATTATAATCTGAGGAAGCTGTTTG	394
	20	TCTGTTCTCTTGGGAGTTGG	AAAATGTAGACTGCCCTAACACTG	281
	21	TTTCCCAGAGAAGAGAATGAGG	AAATGGCATGAGACCCTGAG	374
	22A	CACACCTTCGGACTGTAGCA	TGAGATCCATCACCAAAGCA	998
	22B	CCTACTGACAAACCCCATGC	GAAAGTGTTCGGGGCAGAT	989
HDAC10 (NM_001159286)	1	GAGCCTGCCTAGGTTCTGTG	AACCGCAGACACCTCTGTTC	384
	2	TGAACTGAGCTCCTCATCTGC	GGCCCAGGTTCTTAGGTTTC	233
	3	CCACCATGAATCCCAGGTC	ACGTGCATGTGTGTCTGCAC	227

	4	GTGGGTATGGCCTGCAAAG	GAGTAGGATTTGGGTCACGTC	250
	5	GTGAGAGCTGCCCCTTCC	GTGCTGGCTGGGTTCTCTC	287
	6	GAGAGAACCCAGCCAGCAC	ATCTCACCCCTGGATTTTCC	245
	7	CTCAGGGGTCAGCGTTCTAC	GGGCTTGAGGGTGAACTGT	293
	8	CTCGGCTTCACTGTCAACCT	CTCCTCCTGCCTTCACTGG	341
	9	AAGGCAGGAGGAGGGTTATG	AACCAGAGCCATGTGTGATG	277
	10	GAGGGCTTCTGGGGTCCT	GCTCCCATGCTCCTGACC	213
	11-14	GTGACATCCCATCCCAGTG	CAAGGCAATGTTCACATCCA	968
	15-16	TGGATCTGTTTACCTGGCTTG	TGAGGAAGGCTCCATTCAGT	479
	17	CTCTTCCACCTCTGCCTCTG	ATCATGACAATGCCAGCAAA	240
	18	TTCCTGTCTCCCTGCTCCTA	CACTGGTCCTTTCCCTCGT	343
	19-20	ACGAGGGAAAGGACCAGTG	GTTCCGTGCCCCAGAGTC	480
KRT24 (NM_019016)	1	GGCAGCCTTGAATACACTCTTC	CATCAGGTCCCACTTTGCAT	822
	2	AACACAGGCAACAGCTACCC	TCTCCCGTAACACTTGTTTCA	212
	3	AAACACGGCCAGGTGAGC	CGTGCCAGGCCTATTTACT	461
	4-5	GGCACATTTTCGGAAGTCAT	TGTGCCTAGTGAACCTCTTGG	537
	6	GGCGAAGGTGCACTTCTAGT	AGCCCTGAGTCCCATTTTG	395
	7-8	GAGGCTTTCTCCTGCCTTTG	GATGCCATTTCTTCGCTTGT	546
ACACA (NM_198837)	1	AGAGGGTGCGTTTCAATCAG	CTGCTTTGTGCAATGTCTGG	245
	2	GCTGAGCAAAACAGGAATTGA	TGTGGAAGTCAACTTTTAACACAAA	228
	3	TCTCAAATTGCCTTGCTTCC	TGACATAGGAAAAGGCAAAGC	486
	4	CACAGGATGCAGTTGTGAAAG	TAGGATGCTAGGGAGGCAGA	389
	5	TTGGGGAAGCCAGTTTCTAA	TCCCCCTAAGTTAACATTCTCC	395
	6	TCCCTGACCTTTACGTAGTTTT	CGAAAATGATGCTTTACAAAAATG	299
	7	GGCCAGCCTGGATAGAGAC	AAGAGAGAGAGAGCCCAGCA	276
	8	GGCAACTACATGTGCCAGAA	TTTCAAAAGAGGTAAGTCCCAAA	245
	9	AGAATGCAGAGCAGAAAATTGA	CATCCCTAACACCACCTTGG	289
	10	CATGTGTGGGTGTATGAATGC	TTTTTCTCTGCTAATATGCAGGT	591
	11	TTCTGGACATCTGCTTGTTGA	TGCCATTGTTCTCAGATTGG	395
	12	TGTGCTCTCATGTGGCTAAAA	AGGAGGTGCTTTTCTCTTATCA	397
	13-14	TTAGGGAGTGGCTCCAAAAA	CCATGCTCACACCTTCACTT	850
	15	TTTGTTGCTGAAACAGATTGG	AAAACCCAATCCCAGCATCT	227
	16	TCACAACAAATTTGAGCCTGAT	TGCAGTTTGACTCCCCAAAT	386
	17-18	TCTAGGAAATGCTACAATCAATAATGT	TGGGAAAATCCCAAGAGCTA	834
	19	TGCAGTGAATATGTCTGTGTGTG	CCCAGCCTCCTTTCTGTAAC	385
	20-21	GGTGTGGTCCAACTTAGATGC	GCCAAACAGCTTTCTTTTCC	841
	22	TGGAACCAATTTCCCACGTA	GGAGGATCCAAATAGGAAGGA	373
	23	GAAGATTCCTACTGGTCGTGCT	TTTCCAATTTCTTGAAGTCAAATC	300
	24	TGGAGGTAAATTTGGCCTTT	CCTATAGGGATAGTTTGGGTTGG	246
	25	TTCTTCTCACTGCTCGTCTCA	AGCCCCATTCATCCCATAAT	357
	26	TCCAGCTGGGTTTACTTTGTTT	TGTTCTGAGGAAAACGTGACA	358
	27	TTTATCTGTGATGCCTGCACTT	GGCAGGCCTATTTGATTTTT	463
	28	TCAAATGCCAAATAGTGTCCA	TGACATGGCAGCCAAATAGA	244
	29	CTTCTCTCCCCACAGAGCAC	AAAGAAGTGGGCACCAAAAG	400
	30	TGGAGCTCCTCATGACTGGT	GCTCAGGACTCCCTAGCAGA	246
	31	GCTCTGCAACCTGACCCTAC	ATGGCTATTCGGGAGACCTT	364
	32	GCCACTGGTGATTCTGTTCA	TTCCAGAAAATACTTTGGTGAGG	393
	33	TCTCTTTTTCAGGTATTCTACCAAA	GACATAGCCATAAACTAGCAACTGA	300
	34	AGCCTTACAAGGGCCGAAT	AAGTTGTAGCAGCAGCATAAGAAA	195
	35	GGATTTATGCAGAGAAATAACCA	CTCCCAAAGTGCTGGGATTA	364
	36	GTGATGGTTATGCGGAGGAT	CCAAACCAAACCCTTTCAGA	397

	37	TGTAGCTCATTTTTGTTGCTCA	AGAACAAGAACCTTGATTCAAAA	499
	38	TCTCTTCACCAAGTGCCTTATG	TAAGGGCTCAACAGCCTGAA	391
	39	TTGTCCCTGACTTGTTCCAA	TGAACCAATGGGATTTGTGA	495
	40	GGCATATCTCAGCTACTCAACC	ATTTCTTGCCCTTTGGAGTG	397
	41	TGGAAAAGAAGGCAAAACCA	CCCAACAAGACTACCATGCTC	353
	42	TTTGGCTCTTTAGCAATGTGT	TGGTGATTTTTGAACTTAAACCTCT	500
	43	GCTGTGGAACAAGCTTCATTA	CATCTTCAATGTTCTTCAAGGTT	300
	44	GGCACCAAGCTAAGAAGGAA	TGCTCTCATTTGGGACTGAA	398
	45	GCCTGAATTTTGCATCATTGT	CTGCTTCCTTCCAATCAGGA	397
	46-47	CCCATTTTAGTGCGACTTGTG	TGATTGAAAAGAACGCTAAAAGC	1000
	48	CAGCTGCCTAATGCTTCCAT	CTTGGGCATGGCTATAATCC	390
	49	GGATTGGAAAACAAATGGCTA	GCAATGTTCCTGATGTATTCAC	250
	50	GACAAGGTCAAAGATCGAGGA	GATGTCTCAGAGAGGGCAGAA	463
	51	TATAGGGTGGCCAAACAGGA	CATTCTCCAGGAGGAAACCA	379
	52	GGCCTCATTCTGTTGTCTCC	TTTTTAGGGCTCACTTTCCCTA	379
	53	GTTGAGCTTCCCGTGATTGT	GCTTATGAGCCTGTGTGCAA	323
	54	TACAGCCAGCTCTTGGGTCT	GACTCTTTGCTGGCGAGACT	381
	55	ACGGAGCACACTTTTGGAGT	CCATATCTGTACCTGTGGGTCA	400
	56	GGGCAGTTCTTTAGCTGTCCT	GGTCTCCTGTGCCTTCTCAT	296
	1	TTTGACCCTATTGCTTTTAGCC	CAAGGCACTTCAATTAAGTTTTGA	225
	2	CCTGCATGGTTTTATAGATTCACTT	GAACAAAGAGATGGCATTGGA	393
	3	ACCAATGAGCCTTGCTGACT	CCTGCCTTTTCACACATGAC	336
	4	GAAGTGCTTCCGACGTGAG	TCCACCATGAACATGGAATC	486
	5	GCAGCATGCAGCTCTAAAAA	TGAATCAGGTAGGTGGGTCTC	389
TP63 (NM_001114978)	6	CACCAACATCCTGTTCATGC	GAAACATCCCTGTTGCTGAAA	292
	7	TTGCCCTTTTAGGAGGAAGC	GCAAGGATTTAGAGCACAAAGG	369
	8-9	TGGTAGATCTTCAGGGGACTTT	TCCTTTCCCATTGTCACAGA	1000
	10	TTGCAATTACGGAATCCTCA	GAAAGAGGGTTGCCATACCA	384
	11	GTTTGGTTTGAGGCCATGTT	CCAAGCCACTCCACTTTGAT	398
	12	GCTGGTAGTTTAGGCCCTTG	AACTCCAACCACAGGACTGC	374
	13	TTTCCCTTATCTCGCCAATG	CTACAAGGCGGTTGTCATCA	244
	14	CCAGAGCATCAGGGAATGAT	AGACAAGAGGAAGGGGAGGA	498

Table S5. Reasons for exclusion for the other 44 genes identified by Park et al. [1].

Gene	Reason(s) for Exclusion
BRCA1	Known hereditary cancer gene
BRCA2	Known hereditary cancer gene
ATM	Known hereditary cancer gene
MYH1	Known hereditary cancer gene
MNT	Not a cancer driver gene[a], no disruptive or predicted pathogenic variants in 1006 familial/early-onset CRC patients (Chubb et al.)[b]
KCNG4	Low expression in normal colon mucosa, not a cancer driver gene, high observed vs. expected ratio of LoF variants in controls, disruptive variants in cases << controls (Chubb et al.)
NOP56	Not a cancer driver gene, disruptive variants in cases << controls (Chubb et al.)
TPCN2	Not a cancer driver gene, not involved in colorectal cancer or hereditary CRC pathways/mechanisms
KCNH5	Low expression in normal colon mucosa, not a cancer driver gene, disruptive variants in cases << controls (Chubb et al.)
NIPAL3	Not a cancer driver gene, no disruptive or predicted pathogenic variants in 1006 familial/early-onset CRC patients (Chubb et al.), not involved in colorectal cancer or hereditary CRC pathways/mechanisms
PRPF8	Not a cancer driver gene, not involved in colorectal cancer or hereditary CRC pathways/mechanisms
ANK2	Not a cancer driver gene, predicted pathogenic missense variants in cases << controls (Chubb et al.), not involved in colorectal cancer or hereditary CRC pathways/mechanisms
INO80	Predicted pathogenic missense variants in cases << controls (Chubb et al.)
MYH3	Low expression in normal colon mucosa, not a cancer driver gene, predicted pathogenic missense variants in cases << controls (Chubb et al.)

PATE2	Low expression in normal colon mucosa, not a cancer driver gene, no disruptive or predicted pathogenic variants in 1006 familial/early-onset CRC patients (Chubb et al.)
ABCF3	Not a cancer driver gene, not involved in colorectal cancer or hereditary CRC pathways/mechanisms
GANC	Not a cancer driver gene, disruptive variants in cases << controls (Chubb et al.)
MLH1	Known hereditary cancer gene
GUCY1A3	Predicted pathogenic missense variants in cases << controls (Chubb et al.), not involved in colorectal cancer or hereditary CRC pathways/mechanisms
COL7A1	Not a cancer driver gene, disruptive variants in cases << controls (Chubb et al.)
BEST1	Low expression in normal colon mucosa, not a cancer driver gene, disruptive variants in cases << controls (Chubb et al.)
GLDC	Low expression in normal colon mucosa, not a cancer driver gene, disruptive variants in cases << controls (Chubb et al.)
COLQ	Low expression in normal colon mucosa, not a cancer driver gene, no disruptive or predicted pathogenic variants in 1006 familial/early-onset CRC patients (Chubb et al.)
TULP4	Not a cancer driver gene, not involved in colorectal cancer or hereditary CRC pathways/mechanisms
RPH3AL	Not a cancer driver gene, predicted pathogenic missense variants in cases << controls (Chubb et al.), not involved in colorectal cancer or hereditary CRC pathways/mechanisms
ALDH9A1	Not a cancer driver gene, predicted pathogenic missense variants in cases << controls (Chubb et al.), not involved in colorectal cancer or hereditary CRC pathways/mechanisms
ECEL1	Low expression in normal colon mucosa, not a cancer driver gene
RCL1	Not a cancer driver gene, predicted pathogenic missense variants in cases << controls (Chubb et al.)
ABCC4	Not cancer driver gene, not involved in colorectal cancer or hereditary CRC pathways/mechanisms
MUC17	Not a cancer driver gene, high observed vs. expected ratio of LoF variants in controls, disruptive variants in cases << controls (Chubb et al.)
ALOX12B	Low expression in normal colon mucosa, not a cancer driver gene, disruptive variants in cases << controls (Chubb et al.)
HOGA1	Low expression in normal colon mucosa, not a cancer driver gene, high observed vs. expected ratio of LoF variants in controls
KRT36	Low expression in normal colon mucosa, not a cancer driver gene
BBS1	Not a cancer driver gene, disruptive variants in cases << controls (Chubb et al.)
SCN10A	Low expression in normal colon mucosa, not a cancer driver gene, disruptive variants in cases << controls (Chubb et al.)
THBS3	Not a cancer driver gene, predicted pathogenic missense variants in cases << controls (Chubb et al.), not involved in colorectal cancer or hereditary CRC pathways/mechanisms
RYR1	Low expression in normal colon mucosa, not a cancer driver gene, disruptive variants in cases << controls (Chubb et al.)
USH2A	Low expression in normal colon mucosa, not a cancer driver gene
HK3	Not a cancer driver gene, disruptive variants in cases << controls (Chubb et al.)
ALK	Low expression in normal colon mucosa, predicted pathogenic missense variants in cases << controls (Chubb et al.)
CTSB	Not a cancer driver gene, not involved in colorectal cancer or hereditary CRC pathways/mechanisms
ABCC12	Low expression in normal colon mucosa, not a cancer driver gene, high observed vs. expected ratio of LoF variants in controls, disruptive variants in cases << controls (Chubb et al.)
SLC7A8	Not a cancer driver gene, predicted pathogenic missense variants in cases << controls (Chubb et al.), not involved in colorectal cancer or hereditary CRC pathways/mechanisms
SLC12A4	Not a cancer driver gene, not involved in colorectal cancer or hereditary CRC pathways/mechanisms

[a] Source: https://www.intogen.org, [b] Data source: Rare disruptive mutations and their contribution to the heritable risk of colorectal cancer. Chubb et al. [2].

Table S6. Clinical characteristics, co-segregation data and 2nd hit results of the carriers of the rare, predicted damaging variants identified among the 736 patients included in the study.

**Family ID**	**Variant**	**Proband's Phenotype** *(age at diagnosis)*	**Family History[a] – 1st Degree Affected Relatives** – *(age at diagnosis)*	**Family History[a] – 2nd Degree Affected Relatives** – *(age at diagnosis)*	**Co-Segregation Information**	**2nd Hit Analysis[b]**

**F1**	*NSD1* c.3056G>A; p.(R1019H)	10-20 Ad (50), BC (50)				Not performed
**F2**	*NSD1* c.3089T>C; p.(L1030S)	2 CRC (52, 59), <10 Ad,		BC (46); Lym (9)		CRC: no somatic *NSD1* mutations
**F3**	*NSD1* c.3151G>A; p.(E1051K)	>70 H/SP (55), CRC (55)	13 H/SP (61), 1 Ad (61); 2 polyps (36); CRC (79); BlC (72), LiC (76)	Sto (60)	Carriers: sister, 13 H/SP (61), 1 Ad (61) and son, 2 polyps (36)<br>Non carrier: son, unaffected	CRC: no somatic *NSD1* mutations
**F4**	*HDAC10* c.308C>T; p.(A103V)	BC (26), CRC (35), polyps (35)				Not performed
**F5**	*HDAC10* c.308C>T; p.(A103V)	11 H/SP (63), 11 Ad (63)	LC (79)			Not performed
**F6**	*HDAC10* c.827G>A; p.(R276G)	2 CRC (37, 43), 26 H/SP (37), 1 Ad (37)	Polyps (43); Polyps (62)	CRC (60); PaC (70); PC		Not performed
**F7**	*KRT24* c.130C>T; p.(R44X)	CRC (47)	Lym	Lym; LC; Polyps; BlC; CRC (39); LC		CRC: no somatic *KRT24* mutations
**F8**	*KRT24* c.449G>A; p.(R150H)	OvC (34), <30 H/SP (40), 10 Ad (40), CRC (50)	Polyps (35)	BC (70)		CRC: no somatic *KRT24* mutations in CRC
**F9**	*KRT24* c.1096C>T; p.(R366C)	CRC (40)		CRC (72); EsC		CRC: no somatic *KRT24* mutations
**F10**	*KRT24* c.1096C>T; p.(R366C)	CRC (44)	PC (78); LiC			Not performed
**F11**	*KRT24* c.1096C>T; p.(R366C)	CRC (44)		Leu (1)		CRC: no somatic *KRT24* mutations
**F12**	*KRT24* c.1096C>T; p.(R366C)	CRC (69)	CRC (45)	CRC (80), Sto	Carriers: son, CRC (45)	CRC: no somatic *KRT24* mutations
**F13**	*KRT24* c.1096C>T; p.(R366C)	EC (55), CRC (77)	ThC (4), PC (63), HNC; CRC (48); EC (54)	CRC; LC; PC; BC		Not performed
**F14**	*KRT24* c.1143G>A; p.(M381I)	CRC (35)		Lym		CRC: no somatic *KRT24* mutations
**F15**	*TP63* c.84T>G; p.(H28Q)	CRC (50)	Sto (71)	Sto (43); CRC (70); LiC (50); Sto (40)		Not performed
**F16**	*TP63* c.84T>G; p.(H28Q)	EC (46), CRC (49)	BlC (60), LC (71); OvC (55)	PaC (86), LC (58)		Not performed

**F17**	*TP63* c.1127G>A; p.(R376H)	CRC (56), BC (59)	CRC (56); 2 BC (46, 49); HNC (53)	BC (31); CRC (75); CRC (86); CRC (56); BC		Not performed
**F18**	*TP63* c.1459C>T; p.(R487C)	5 CRC (39), 11-20 Ad (39)				Not performed

[a] Individual cancer-affected family relatives are separated by semicolons. [b] Mutational analysis of the corresponding gene in the tumor was performed by Sanger Sequencing. Chromatograms did not show evidence of LOH in any of the tested tumors for the corresponding gene, when assessing the allele peak height corresponding to the germline variant in tumor DNA compared to blood DNA. Abbreviations: Ad., adenoma; BC, breast cancer; BlC, bladder cancer; BrC, brain cancer; CRC, colorectal cancer; DCy, desmoid cyst; EC, endometrial cancer; EsC, esophageal cancer; GC, gastric cancer; HNC, head and neck cancer; H/SP, hyperplastic/serrated polyps; OC, oral cancer; OvC, ovarian cancer; LarC, laryngeal cancer; LC, lung cancer; Leu, leukemia; LiC, liver cancer; Lym, lymphoma; PaC, pancreatic cancer; PC, prostate cancer; Sto, stomach cancer; ThC, thyroid cancer.

Table S7. Case-control analysis of the germline variants identified in the five selected candidate genes, considering the gnomAD non-Finnish European (NFE), non-cancer subpopulation as controls.

**Gene**	**Cohort or Study**	**Disruptive Alleles**		**Disruptive, Splice-Site, Start-Loss, Predicted Pathogenic Missense (REVEL>0.4)**	
		**n/ Total Alleles (%)**	**OR (95%CI); p-value**	**n/ Total Alleles (%)**	**OR (95%CI); p-value**
NSD1	Controls (gnomAD NFE non-cancer)	7/118190 (0.01%)		281/118190 (0.24%)	
	Familial/EOCRC				
	Zhunussova et al [3]	0/250 (0.00%)		1/250 (0.4%)	
	Chubb et al [4]	1/2012 (0.05%)		8/2012 (0.40%)	
	Current study	0/930 (0.00%)		0/930 (0.00%)	
	Subtotal	1/3192 (0.03%)	5.29 (0.12-41.21); p=0.19	9/3192 (0.28%)	1.19 (0.54-2.29); p=0.58
	Polyposis (current study)	0/542 (0.00%)	0 (0.00-151.78); p=1	1/542 (0.18%)	0.78 (0.02-4.38); p=1
	TCGA CRC patients	0/1086 (0.00%)	0.00 (0.00-75.56); p=1	2/1086 (0.18%)	0.77 (0.09-2.83); p=1
	**TOTAL**	**1/4820 (0.02%)**	**3.50 (0.08-27.28); p=0.27**	**12/4820 (0.25%)**	**1.05 (0.53-1.86); p=0.88**
HDAC10	Controls (gnomAD NFE non-cancer)	138/118190 (0.12%)		432/118190 (0.37%)	
	Familial/EOCRC				
	Chubb et al [4]	3/2012 (0.15%)		6/2012 (0.30%)	
	Current study	0/930 (0.00%)		0/930 (0.00%)	
	Subtotal	3/2942 (0.10%)	0.87 (0.18-2.61); p=1	6/2942 (0.20%)	0.56 (0.20-1.22); p=0.21
	Polyposis (current study)	0/542 (0.00%)	0 (0.00-5.92); p=1	3/542 (0.55%)	1.52 (0.31-4.49); p=0.46
	TCGA CRC patients	0/1086 (0.00%)	0.00 (0.00-2.95); p=0.64	3/1086 (0.28%)	0.76 (0.15-2.23); p=1
	TOTAL	3/4570 (0.07%)	0.56 (0.11-1.68); p=0.50	12/4570 (0.26%)	0.72 (0.37-1.27); p=0.31
KRT24	Controls (gnomAD NFE non-cancer)	92/118190 (0.08%)		555/118190 (0.47%)	
	Familial/EOCRC				
	Chubb et al [4]	2/2012 (0.10%)		6/2012 (0.30%)	
	Current study	1/930 (0.11%)		7/930 (0.75%)	
	Subtotal	3/2942 (0.10%)	1.31 (0.27-3.95); p=0.50	13/2942 (0.44%)	0.94 (0.50-1.62); p=1
	Polyposis (current study)	0/542 (0.00%)	0.00 (0.00-8.94); p=1	1/542 (0.18%)	0.39 (0.01-2.20); p=0.53
	TCGA CRC patients	2/1086 (0.18%)	2.37 (0.28-8.82); p=0.21	**13/1086 (1.20%)**	**2.57 (1.35-4.45); p= 0.002**

	TOTAL	5/4570 (0.11%)	1.41 (0.45-3.40); p=0.41	27/4570 (0.59%)	1.26 (0.82-1.86); p=0.23
ACACA	Controls (gnomAD NFE non-cancer)	19/118190 (0.02%)		334/118190 (0.28%)	
	Familial/EOCRC				
	Thutkawkorapin et al [5]	0/102 (0.00%)		1/102 (0.98%)	
	Chubb et al [4]	1/2012 (0.05%)		4/2012 (0.20%)	
	Current study	0/930 (0.00%)		0/930 (0.00%)	
	Subtotal	1/3044 (0.03%)	2.04 (0.05-12.86); p=0.40	5/3044 (0.16%)	0.58 (0.19-1.37); p=0.29
	Polyposis (current study)	0/542 (0.00%)	0.00 (0.00-46.89); p=1	0/542 (0.00%)	0.00 (0.00-2.42); p=0.41
	TCGA CRC patients	1/1086 (0.09%)	5.73 (0.14-36.16); p=0.17	5/1086 (0.46%)	1.63 (0.53-3.86); p=0.24
	TOTAL	2/4672 (0.04%)	2.66 (0.30-11.05); p=0.19	10/4672 (0.21%)	0.76 (0.36-1.41); p=0.48
TP63	Controls (gnomAD NFE non-cancer)	1/118190 (0.001%)		352/118190 (0.30%)	
	Familial/EOCRC				
	Chubb et al [4]	0/2012 (0.00%)		4/2012 (0.20%)	
	Current study	0/930 (0.00%)		3/930 (0.32%)	
	Subtotal	0/2942 (0.00%)	0.00 (0.00-1529.34); p=1	7/2942 (0.24%)	0.80 (0.32-1.66); p=0.73
	Polyposis (current study)	0/542 (0.00%)	0.00 (0.00-7507.02); p=1	1/542 (0.18%)	0.62 (0.02-3.48); p=1
	TCGA CRC patients	0/1086 (0.00%)	0.00 (0.00-3980.48); p=1	0/1086 (0.00%)	0.00 (0.00-1.15); p=0.08
	TOTAL	0/4570 (0.00%)	0.00 (0.00-992.99); p=1	8/4570 (0.18%)	0.59 (0.25-1.17); p=0.16

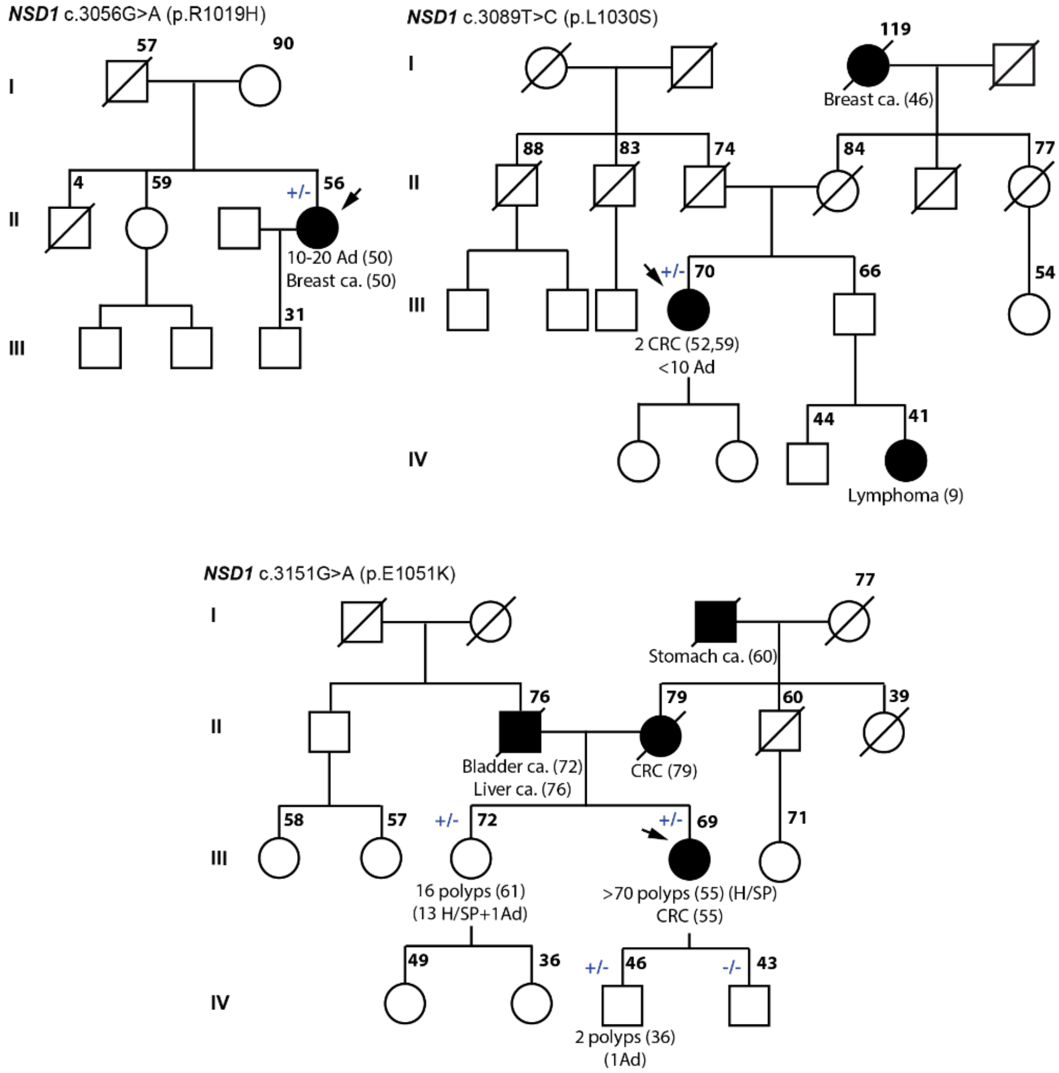

Figure S1. Pedigrees of the families carrying *NSD1* rare germline (predicted) damaging variants. Filled black symbol, affected with cancer; black arrow, index case. Ages at information gathering or at death, when available, are indicated on the top-right corner, and ages at cancer diagnosis, after tumor type. +/- depicts heterozygous carrier status, and -/- non carrier of the corresponding *NSD1* variant.

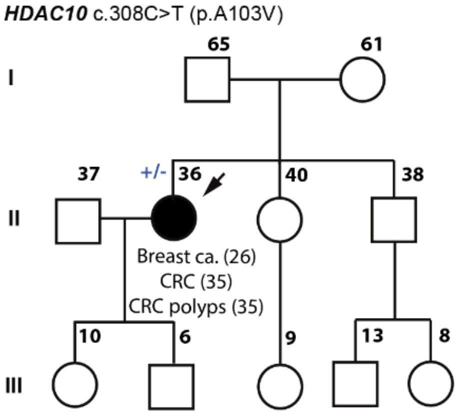

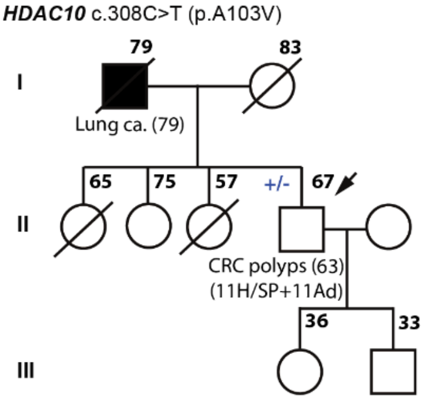

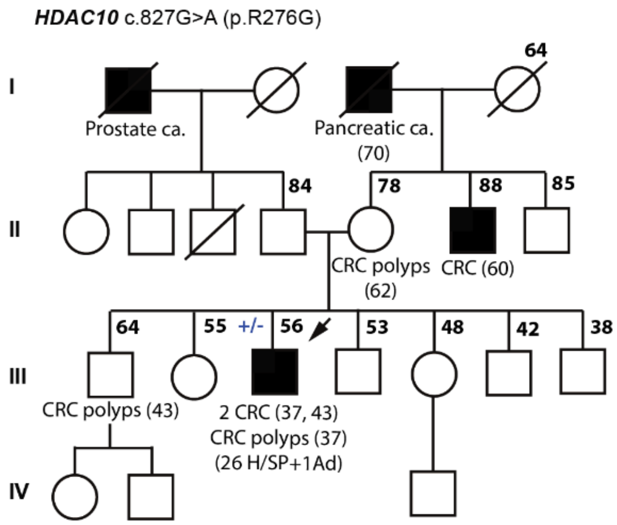

Figure S2. Pedigrees of the families carrying *HDAC10* rare germline variants from the cohort analyzed in the current study. Filled black symbol, affected with cancer; black arrow, index case. Ages at information gathering or at death, when available, are indicated on the top-right corner, and ages at cancer diagnosis, after tumor type. +/- depicts heterozygous carrier status of the corresponding *HDAC10* variant.

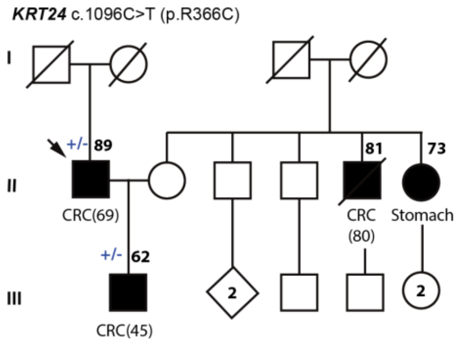

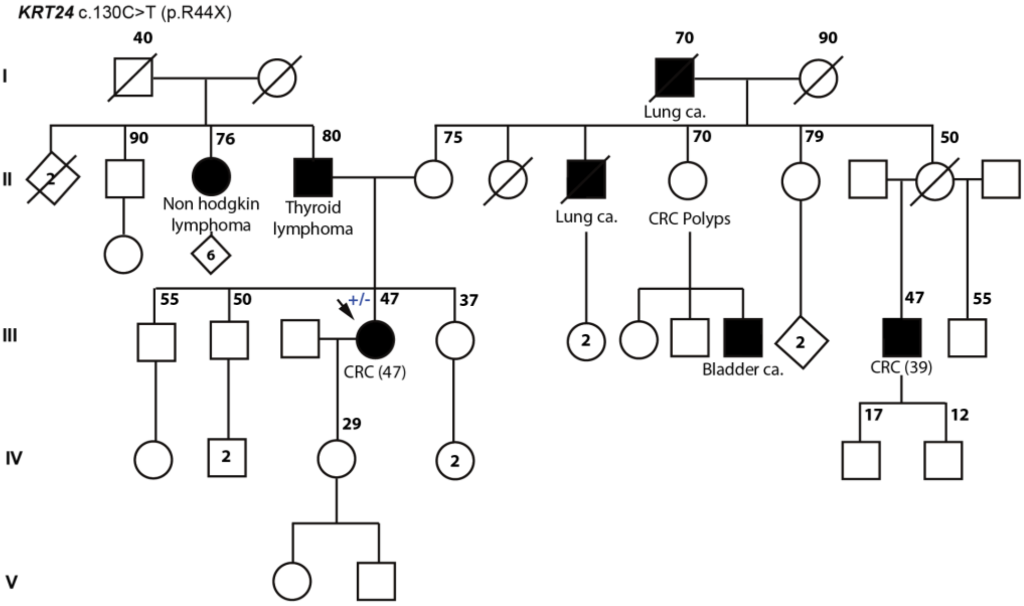

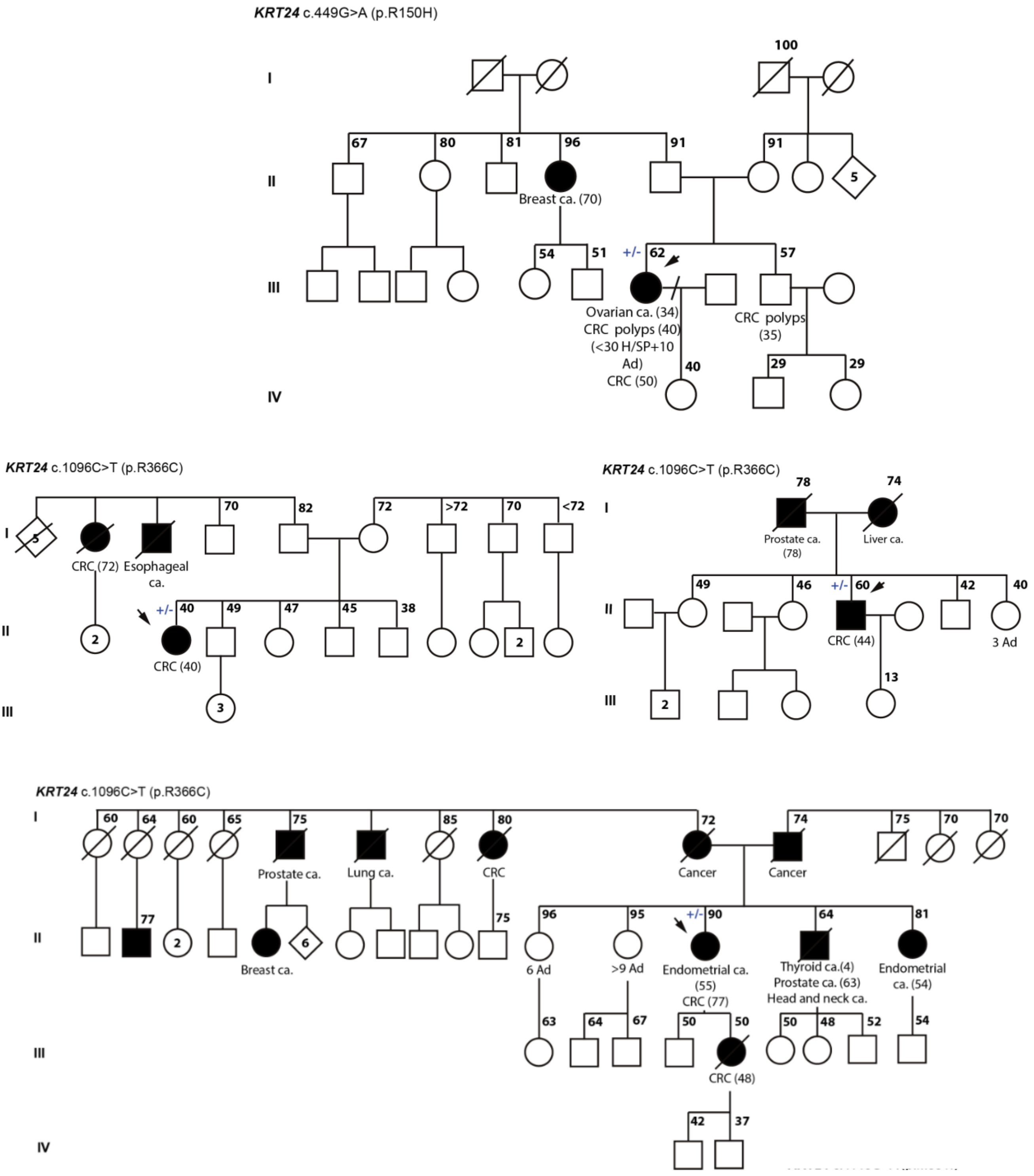

KRT24 c.449G>A (p.R150H)
Breast ca. (70)
Ovarian ca. (34)
CRC polyps (40)
(<30 H/SP+10 Ad)
CRC (50)
CRC polyps (35)
KRT24 c.1096C>T (p.R366C)
CRC (72)
Esophageal ca.
CRC (40)
KRT24 c.1096C>T (p.R366C)
Prostate ca. (78)
Liver ca.
CRC (44)
3 Ad
KRT24 c.1096C>T (p.R366C)
Prostate ca.
Lung ca.
CRC
Cancer
Cancer
Breast ca.
6 Ad
>9 Ad
Endometrial ca. (55)
CRC (77)
Thyroid ca.(4)
Prostate ca. (63)
Head and neck ca.
Endometrial ca. (54)
CRC (48)

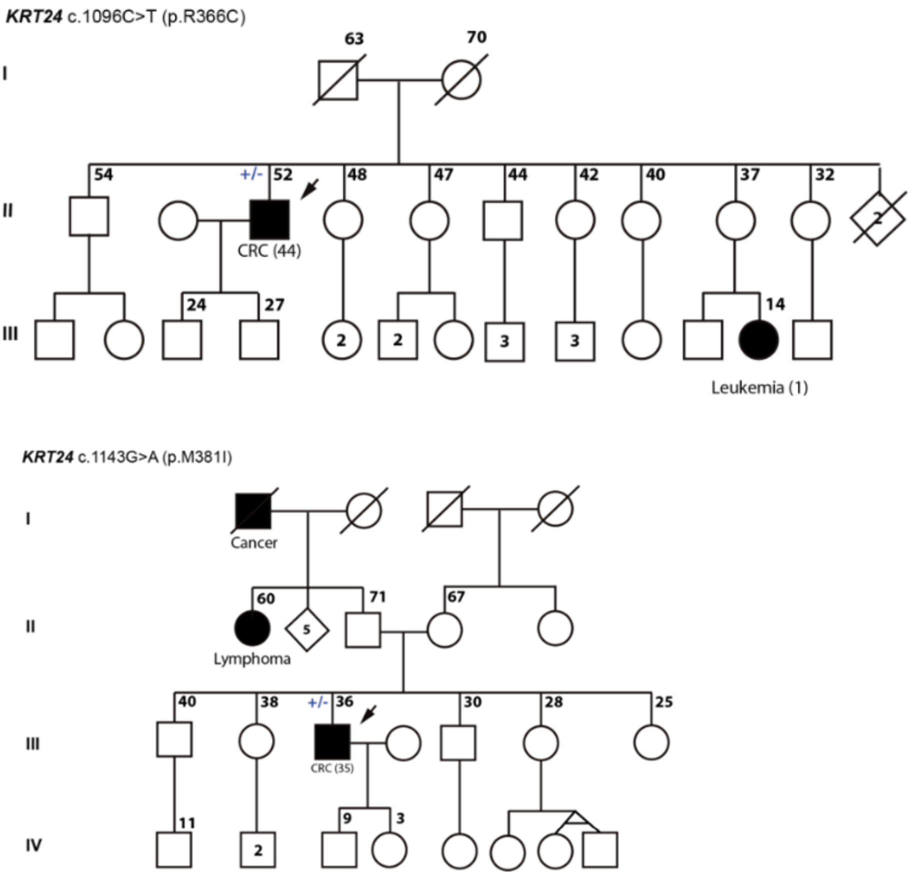

Figure S3. Pedigrees of the families carrying *KRT24* rare germline variants from the cohort analyzed in the current study. Filled black symbol, affected with cancer; black arrow, index case. Ages at information gathering or at death, when available, are indicated on the top-right corner, and ages at cancer diagnosis, after tumor type. +/- depicts heterozygous carrier status of the corresponding *KRT24* variant.

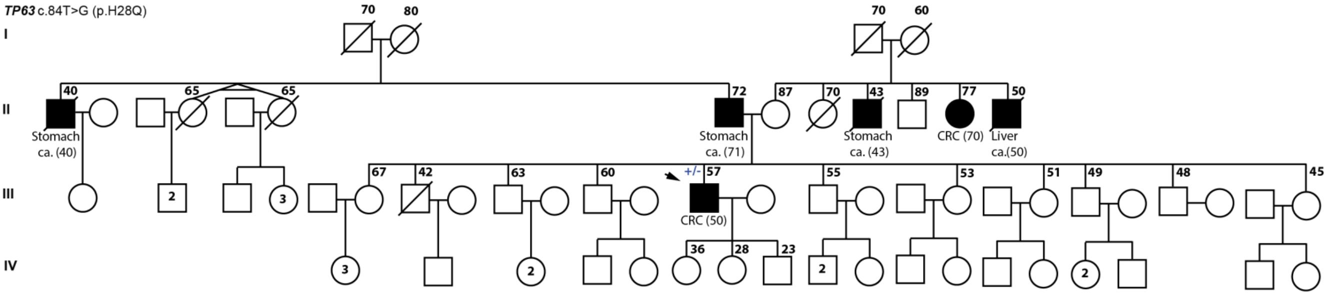

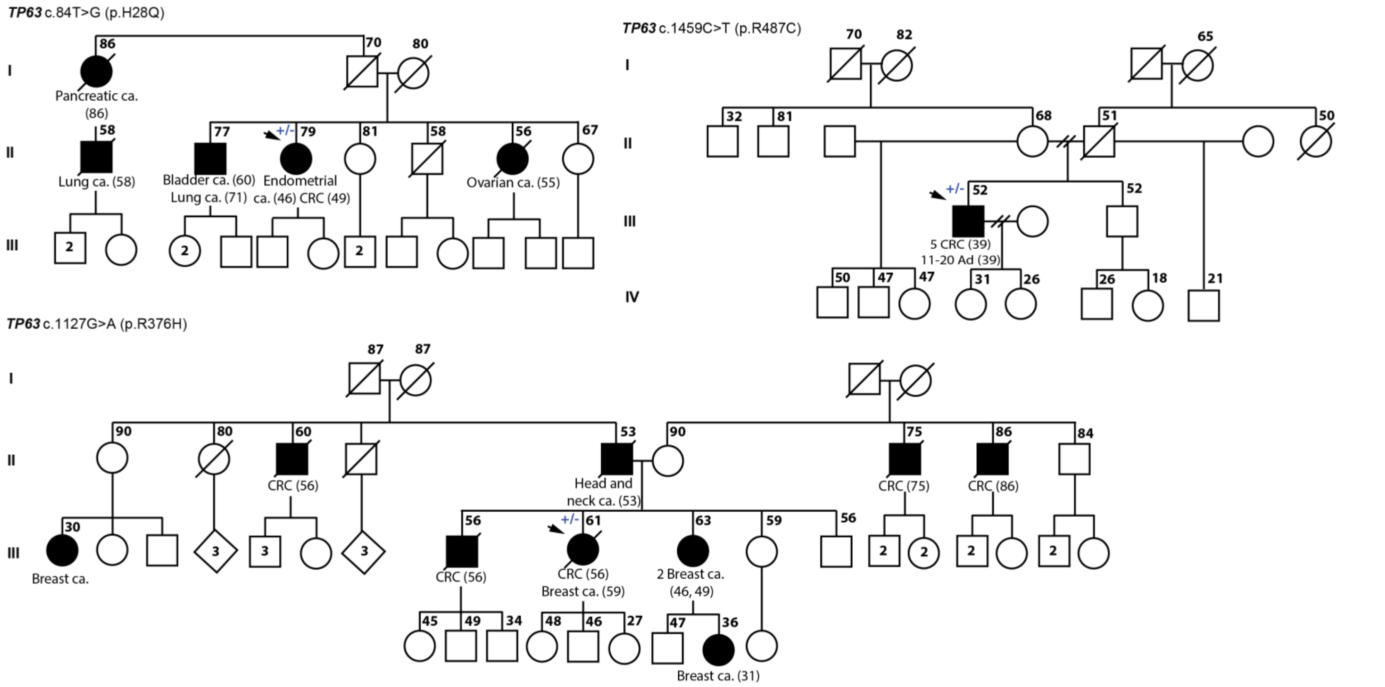

Figure S4. Pedigrees of the families carrying *TP63* rare germline variants from the cohort analyzed in the current study. Filled black symbol, affected with cancer; black arrow, index case. Ages at information gathering or at death, when available, are indicated on the top-right corner, and ages at cancer diagnosis, after tumor type. +/- depicts heterozygous carrier status of the corresponding *TP63* variant.

REFERENCES

1. Park, S.; Supek, F.; Lehner, B. Systematic discovery of germline cancer predisposition genes through the identification of somatic second hits. *Nat. Commun.* **2018**, *9*, 2601. https://doi.org/10.1038/s41467-018-04900-7.
2. Chubb, D.; Broderick, P.; Dobbins, S.E.; Frampton, M.; Kinnersley, B.; Penegar, S.; Price, A.; Ma, Y.P.; Sherborne, A.L.; Palles, C.; et al. Rare disruptive mutations and their contribution to the heritable risk of colorectal cancer. *Nat. Commun.* **2016**, *7*, 11883. https://doi.org/10.1038/ncomms11883.
3. Zhunussova, G.; Afonin, G.; Abdikerim, S.; Jumanov, A.; Perfilyeva, A.; Kaidarova, D.; Djansugurova, L. Mutation Spectrum of Cancer-Associated Genes in Patients With Early Onset of Colorectal Cancer. *Front. Oncol* **2019**, *9*, 673. https://doi.org/10.3389/fonc.2019.00673.
4. Chubb, D.; Broderick, P.; Dobbins, S.E.; Houlston, R.S. CanVar: A resource for sharing germline variation in cancer patients. *F1000Res.* **2016**, *5*, 2813. https://doi.org/10.12688/f1000research.10058.1.
5. Thutkawkorapin, J.; Lindblom, A.; Tham, E. Exome sequencing in 51 early onset non-familial CRC cases. *Mol. Genet. Genomic Med.* **2019**, *7*, e605. https://doi.org/10.1002/mgg3.605.
